# Program participation in a targeted land distribution program and household outcomes: evidence from Vietnam

**DOI:** 10.1007/s11150-017-9390-0

**Published:** 2017-09-20

**Authors:** Dwayne Benjamin, Loren Brandt, Brian McCaig, Nguyen Le Hoa

**Affiliations:** 1 University of Toronto, Toronto ON, Canada; 2 Wilfrid Laurier University, Waterloo ON, Canada; 3 Institute of Policy and Strategy for Agriculture and Rural Development (IPSARD, Hanoi), Hanoi, Vietnam

**Keywords:** Land policy, Program evaluation, Vietnam, Ethnic minority households, Q15, I3, O12, O13

## Abstract

We estimate whether a land reform program led to higher incomes for ethnic minority households. In 2002, in the Central Highlands of Vietnam, Program 132 directed the transfer of farm land to ethnic minority households that had less than one hectare of land. Using the 2002 Vietnam Household Living Standards Survey as a baseline, in 2008 we resurveyed over one-thousand households to provide a retrospective evaluation of the impact of their participation in Program 132. Contrary to official reports, our findings show that there was considerable deviation from the planned program parameters: many eligible households did not receive land, while ineligible households often did. We estimate that beneficiaries of the program in the province of Kon Tum experienced increases of household income largely in line with what one would expect from a small plot of poor farm land. Outside Kon Tum, where participation rates were substantially lower, the story is more mixed, and household incomes did not improve with program participation. Overall, our results underscore the limitations of simple transfers of land as a mechanism for improving the living standards of ethnic minorities. Our results also show the significant gap that can exist between program design and decentralized implementation. We discuss the potential implications for program evaluation.

## Introduction and overview

In 2002, the government of Vietnam announced a plan to redistribute land to land-poor ethnic minority households in the Central Highlands (CH) region. This was prompted by long simmering ethnic conflict that boiled over between indigenous minorities, and the more recently settled Kinh, Vietnam’s largest ethnic group. Policymakers hoped that by granting secure access to agricultural land, ethnic minority households could better participate in the rapidly expanding commercial agriculture sector, and thereby improve their poor economic status. Program 132 as drafted in Hanoi covered three single-spaced pages and defined precise eligibility criteria: Ethnic minority households with less than one hectare of farmland would be topped up to one hectare, subject to local land availability.

According to official reports and our own interviews with local officials, the policy was executed in line with the original pronouncement. One-sixth of ethnic minority households, and over half of those households deemed eligible, were reported to receive land, with an average transfer of about half a hectare. Program participation rates were especially high in the more sparsely populated province of Kon Tum, where almost forty percent of ethnic minority households received land.

We investigate whether participation in Program 132 led to higher incomes for ethnic minority households, and, if so, how effective the land transfers were for improving low living standards. We used the 2002 Vietnam Household Living Standards Survey (VHLSS) as a baseline and then, in 2008, resurveyed 1126 households within fifty communes in the Central Highlands. This provides us with detailed household-level data on agricultural land holdings and economic outcomes before and after the implementation of Program 132.

We paint a rich picture of the “before and after” economic outcomes of minority households in the Central Highlands, and draw plausible conclusions about the causal impact of Program 132 on the living standards of these households. First, “treatment” through the program may not have been as high as officially reported and implementation details varied significantly across communes. In particular, the type of land (annual, perennial, or a combination) and area used to define eligibility differed across communes, with many communes using a lower threshold than stipulated in the program. Second, land was frequently given to apparently ineligible households. While land was transferred to minority households—there appears to have been no leakage to Kinh households—it was not targeted to those with the least amount of land or lowest incomes. Third, in Kon Tum province, we find that households granted access to land classified as annual (used for growing annual crops, like cassava) saw their crop income increase in line with the returns to this type of land. Outside Kon Tum, the effects on income were negligible, reflecting lower treatment rates and lags in the maturity of perennial crops like coffee and cashews. Overall, the program did little to improve the relative position of minority households: There is only so much income that a half-hectare of land can generate.

Our results indicating the weak link between program eligibility and actual participation add to the growing literature that underscores the challenges of targeting the poor for transfers, even on as simple a proxy as land holdings. While it is true that any transfer to minority households in the Central Highlands (even a random one) is probably welfare improving, targeting on the basis of land alone may lead to less efficient transfers. First, land is only loosely related to household per capita income, and far from sufficient to generate agricultural income. Second, even well-executed targeted programs have errors (see Alatas et al. 2012). Moreover, a strict adherence to a targeting regime may be unpopular, especially when only a subset of households benefit. Community-based, or participatory schemes, that allow for local input on the targeting can be more effective and politically sustainable (see Alatas et al. 2012, and Karlan and Thuysbaert 2016). To be clear, the loose implementation of Program 132 from Hanoi through the commune level may have had little to do with these considerations. While there is no evidence that corruption played a role (e.g., as in Niehaus and Sukhtankar 2013), a variety of local conditions and incentives may result in poor targeting.

The impact of the policy was determined as much by variation in its implementation across communes, as by the value of land itself. Moreover, program implementation and the value of land were probably interconnected as communes where land was scarce (and more valuable) could transfer less land than those where land was plentiful (and less valuable). The challenges to impact evaluation in this context arise at least as much from endogenous variation of treatment across communes as deviations from intended treatment within communes. We believe our analysis is informative for discussions about program evaluation. In theory, the program had a clearly defined rule for defining an eligible household (less than a hectare of land) and the corresponding amount of land the household should receive. However, in practice, local implementation varied significantly from these national guidelines.

What does this teach us about program evaluation? Consider a well-designed randomized control trial (RCT). In the current context, it randomly assigns some communes to be treated by the programs and others to not be (i.e., randomization would be at the level of the commune, as in Alatas et al. 2012). This allows for a straightforward calculation of the average impact across communes. Additionally, it enables us to say something about the impact of constraints and incentives faced by local leaders when implementing the program, depending on how treatment varied across communes. Still as emphasized by Deaton (2010), Ravallion (2008, 2009), and others, while internal validity is assured, external validity remains a serious concern. Indeed, our results suggest that officials commonly reported to their supervisors that the program was implemented as planned by the national government despite large deviations in some communes. As such, the actions of local officials may be different under a closely scrutinized RCT than when being watched less closely, say, during a large scale up of the program based on an initial RCT evaluation.

Sometimes, however, experiments are not feasible. Instead, even if there are theoretical limits to causal inference, there is a value in documenting what happened when a policy was implemented. A necessary condition for this is that there are regular, well-designed household surveys like the VHLSS. This survey grew out of the larger living standards measurement study project, of which Angus Deaton was a key and early contributor. Indeed, the methods we employ here address the questions raised by Ashenfelter, Deaton, and Solon (1986) in discussing the relative merits of collecting cross-section and panel household data in developing countries. Our research is predicated on the existence of the repeated VHLSS cross-sections, while creating a purpose-built panel data set to measure changes in land holding, income, and program participation. The combination of unintended program implementation, and the returns to regular data collection also highlight the “Monitoring” part of Monitoring and Evaluation (e.g., Clark, et al. 2004). Regular observation of local (institutional) implementation of a program allows for a much more accurate program evaluation. Without the monitoring, it is challenging to interpret the results of even the most plausible program evaluation.

Our analysis is also related to the scarce international evidence on the impact of land reform. For example, Keswell and Carter (2014) evaluate land redistribution in South Africa, and find that the initial impact (1 year) was negative, but ultimately large, peaking after 3 years. While our effects are much smaller, timing may also matter. Beneficiaries outside of Kon Tum, where perennials are more important, did not experience an increase in income, possibly because of the time it takes for perennial crops like cashews and coffee to mature, and generate income.

The remainder of our paper is as follows. In the next section, we provide a more detailed overview of Program 132, as well as a description of the economic conditions of minority households in the Central Highlands in 2002. After describing our sampling strategy and new data set, we then describe patterns of program participation (treatment): Who received land from Program 132, and how did this relate to eligibility as predicted in 2002? We compare results from different data sources, and show the poor targeting performance of the program. We then explore the potential impact of treatment, first on household land holdings, and second on household income, including a detailed discussion of the evolution of minority household incomes between 2002 and 2008. To do this, we estimate the value of a hectare of land to a minority household, and compare this to the estimated effect of program participation. In our final section, we draw together our conclusions, and potential lessons from this exercise.

## Background

### Ethnic minorities in Vietnam

Despite rising absolute living standards, minorities lag significantly behind the Kinh. For example, between 1998 and 2010 per capita consumption rose 7.4% for minorities, but was a full 2.0 percentage points slower than for Kinh households (World Bank 2013). The gap grew through 2014 (Benjamin, Brandt, and McCaig 2017). However, the rapid growth experienced by minorities as a whole hides significant differences in outcomes across minority groups. For example, many minority groups in the Central Highlands, such as the Xo-Dang and Gia Rai, experience household rates of poverty of more than 80 percent (World Bank 2013). Non-monetary outcomes, such as education and nutrition, show similar disparities (Baulch et al. 2010).

The differences in outcomes between Kinh and minorities and possible explanations for them have been extensively studied.^1^ Both World Bank (2013) and Baulch et al. (2010) identify important differences in endowments between minorities and Kinh. Minorities have lower levels of education, have poorer quality land, face greater barriers to accessing credit, and are more isolated than Kinh households. These lead to differences in employment and income-generating opportunities across the two groups, as minorities are much less likely to seek off-farm employment, or be involved in non-farm businesses (Baulch et al. 2010). A decomposition of the differences in per capita expenditure suggest that between one-third and half of the gap is due to differences in endowments and other household and community characteristics (Baulch et al. 2010). Given the importance of agriculture to minority households, agricultural land is potentially a key endowment and has been a source of conflict between ethnic minorities and Kinh in the Central Highlands region.

### Land redistribution in the Central Highlands

In 2001, and then again in 2004, Vietnam’s Central Highlands provinces were disrupted by protests by ethnic minorities.^2^ There have been numerous assessments in the press, by NGOs as well as by academics of the complex economic, political and social forces underlying the unrest.^3^ Issues of religious freedom often come up, but at the core appears to be economic factors, especially those related to land, that have been playing out for several decades. Disruption of ethnic minorities’ customary land rights and traditional forms of agriculture following the end of the Vietnam War in 1975; waves of migration into the region by Kinh and other ethnic minority households, accompanied by resettlement of ethnic minority within the region; and commodity boom-bust cycles beginning in the mid-1990s, have all contributed to perceptions of the growing economic marginalization of ethnic minority households in the region, and a widening gap with the Kinh in the region.

To help address these concerns, in late 2002 the central Government of Vietnam implemented Program 132. The program was designed to redistribute farmland to land-scarce ethnic minority households in the Central Highlands, to improve their lives, enhance the development and ensure the security in Central Highland regions (Article 1 of Decision 132).^4^ For a variety of historical reasons, many minority households had only tenuous claims on plots of agricultural land, and the government hoped that by providing secure long-term access to land, households would invest in the land, and be better able to earn a livelihood farming. The policy objective was clearly stated. Farm households should have a minimum of 1.0 hectares of agricultural land, with some adjustments made for paddy land. The minimum distribution of agriculture land and residential land for each household is 1 hectare of terrace land or 0.5 hectare of paddy land (single crop) or 0.3 hectare of paddy land (double crop) and 400 $m^2$ for residential land (Article 2 of Decision 132). As paddy land is almost non-existent in our sample, we set aside these distinctions for the remainder of the paper. Households were granted full use rights to the land, with the restriction that they could not sell or mortgage the land for 10 years. They were expected to farm the land.

Implementation of Program 132 was delegated to lower levels of government, with responsibility spread across several ministries. The provincial Ministries of Agriculture and Rural Development (MARD) had primary responsibility, shared with Provincial Peoples Committees, Provincial Ministries of Finance (to oversee budgetary issues), Provincial Ministries of Natural Resources and the Environment (to oversee compliance with environmental regulations, especially pertaining to forests), and local Committees for Ethnic Minorities. From the provincial level, implementation was further delegated to the district, and ultimately, the commune level. Land redistribution was subject to local land availability, and local needs (unlike money, land cannot be shifted from one place to another). Whatever elements of common program design existed would be subject to local constraints in implementation. Commune governments were typically responsible for assessing eligibility, and the actual distribution of land. In some communes, the new land was assigned by commune officials, while in others, households drew lots to choose new plots.

The sources of available land also varied. In some communes, land was available from adjacent agro-forest plantations, typically operated by state-owned forestry companies. Some communes also had publicly managed land that could be made available to households. Land could also be purchased from other households by the government for redistribution. If in compliance with environmental regulations, forestland could also be transferred to households. Finally, land reclaimed from free land, treeless hills, and non-used land, in other words, land with nebulous status, could also be transferred to households. The transferred land need not be plough-ready, and households were given up to 4 million VND (about US$235) to cover the costs of land reclamation.

Shortly after Program 132 was announced, in 2004 Program 134 was implemented. Program 134 essentially extended Program 132 to ethnic minority households outside the Central Highlands. One key difference was that the land thresholds and redistribution targets were not as high as in Program 132 (i.e., 0.5 hectare instead of 1.0 hectare). In addition, Program 134 added housing and drinking water to the existing Program 132 infrastructure. While we do not evaluate the housing and water dimensions of Program 134, because of the overlap in program administration, we treat Programs 132 and 134 as a package, though referring primarily to Program 132, as its parameters were most relevant for farmland in the Central Highlands.

There have been a number of official assessments of Programs 132 and 134. These draw on a combination of commune, district and provincial-level reports. The main objective of these assessments was to account for the extent of land redistribution, and tally how many households benefited from the program. Of these, MARD (2006) is probably the most comprehensive. These reports paint a mixed picture of the extent and intensity of treatment (program participation). We summarize the provincially reported treatment rates in Table 1. The bottom row shows the number of households, and corresponding treatment rates for the entire Central Highlands. Of over 250,000 ethnic minority households, 28.3% were deemed eligible for the program. Unfortunately, eligibility is not explicitly defined in the report, so it is unclear what this means. What is clearer is the reported number of treated households who received land, over 43,000 households. This represents sixty percent of eligible households, and 17 percent of all minority households in the region. These numbers indicate widespread program participation. The total amount of land transferred was almost 21,000 hectares, which implies an average redistribution of almost a half-hectare of land per household.

**Table 1:** Provincial reports of household (HH) program 132 participation (treatment rates)

Province	Ethnic minority HH	“Eligible” HH	HH received land	% Of ethnic minority eligible	% Of minority “Treated”	% Of “Eligible” treated	Land received (Ha.)	Land received per HH
Kon Tum	34,488	15,678	12,836	45.5	37.2	81.9	5793	0.45
Gia Lai	80,208	16,170	12,596	20.2	15.7	77.9	4083	0.32
Dak Lak	100,353	20,981	8202	26.3	10.3	39.1	4556	0.56
Dak Nong	—	2120	2120	—	—	100.0	1283	0.61
Lam Dong	38,700	16,856	7519	43.6	19.4	44.6	5026	0.67
Total	253,749	71,805	43,273	28.3	17.1	60.3	20,741	0.48

*Notes:* (1) Source: Authors’ tabulations based on official Provincial Reports of Program 132; (2) “Eligibility” is taken as defined in the official reports. (3) The “Total” numbers for the Central Highlands also includes the small number of households that are in the newly created province of Dak Nang (which used to be part of Dak Lak). In Dak Nong, there were 2120 minority households, all of which received land under the program

The other rows of Table 1 report comparable numbers for each province. There is significant heterogeneity across provinces in program implementation, with the highest percentage of eligible households in Kon Tum, followed by Lam Dong. Kon Tum also reported the highest percentage of ethnic minority households being treated (37.2%) and the highest percentage of eligible households that were treated (81.9%). Neighboring Gia Lai province had the next highest rate of treatment for eligible household (77.9%). In contrast, less than half of eligible households in Dak Lak or Lam Dong received land. The main reason for the variation of treatment rates of eligible households appears to have been a shortage of available land. Irrespective of province, those households that were treated received on average slightly less than half a hectare of land.

In summary, Table 1 suggests that Program 132 succeeded in distributing a considerable amount of land to minority households. Underlying the treatment rates is an important assumption: While some eligible minority households did not receive land, no Kinh households received land. The provincial, aggregate data do not permit this sort of evaluation. Nor is there any evidence in these numbers that the program actually helped minority household living standards. As emphasized by Roumasset and Lee (2007), despite the attraction of “lump sum” redistribution of endowments suggested by the Second Welfare Theorem, land reform need not yield the predicted benefits to its beneficiaries. To evaluate those questions, we designed a household survey to assess linkages between program participation and household outcomes.

## Data and initial conditions

The 2002 VHLSS provides an excellent baseline survey of households just prior to the implementation of Program 132. For our purposes, the VHLSS has detailed information on household land holdings and ethnicity, the key determinants of program eligibility, as well as a rich array of pre-program outcomes like household income. For the post-survey, we sought to resurvey 1250 households: All of the original sample of 25 households per commune, drawn from 50 out of 120 Central Highlands communes in the 2002 VHLSS. We skewed our selection of communes towards maximizing the number of potentially treated ethnic minority households, based on observed land holdings in 2002. For administrative reasons, we also excluded all communes that in 2002 were in Dak Lak, but became part of Dak Nak province after 2003. Relative to their share of the rural population in the Central Highlands, we over-sampled in Kon Tum and Gia Lai, and under-sampled in Dak Lak and Lam Dong, as our objective was to maximize the number of households that would have been eligible for treatment. For households, we adapted the full VHLSS questionnaire, with additional modules on Program 132 and 134 participation. We also conducted surveys at the commune and district level, with modules added relating to 132 and 134 implementation. The resulting household survey, the *Central Highlands Vietnam Living Standards Survey* (CHVLSS) included 1126 panel households (i.e., households surveyed in both 2002 and 2008) with complete information.^5^

The objective of Program 132, and to a slightly less extent 134, was to redress differences in land endowments between ethnic minority and Kinh households in the Central Highlands through allocation of land to the former. Thus, it is useful to examine differences in landholdings between the two types of households before the policies were implemented, which we report in Table 2. The four Central Highland provinces we examine are not identical in this regard. Indeed, for reasons that will soon become clear, we separate results for the Central Highlands into (1) Kon Tum and (2) the Central Highlands outside Kon Tum. Altogether, we have data on 230 households in Kon Tum, of which 207 are ethnic minority, and 896 outside Kon Tum, of which 629 are ethnic minority.

**Table 2:** Comparisons of households in 2002: ethnic minority vs. non-minority; Kon Tum vs. Central Highlands (ch) outside Kon Tum

	Kon Tum		CH (Non-Kon Tum)	
	Non-minority	Minority	Non-minority	Minority
Average household land				
Annual land (Ha.)	1.43	1.06	0.31	0.77
Perennial land (Ha.)	0.55	0.06	0.49	0.47
Agricultural land (annual + perennial, Ha.)	1.97	1.12	0.80	1.23
Forestry land (Ha.)	0.03	0.76	0.02	0.03
Land distribution				
Proportion of households with:				
Annual land = 0	0.00	0.00	0.44	0.15
Annual land > 0 & < 0.5	0.04	0.12	0.35	0.27
Annual land $\ge$ 0.5 & < 1.0	0.30	0.41	0.10	0.25
Annual land $\ge$ 1.0	0.65	0.47	0.11	0.33
Agricultural land = 0	0.00	0.00	0.10	0.01
Land > 0 & < 0.5	0.04	0.11	0.31	0.17
Annual land $\ge$ 0.5 & < 1.0	0.09	0.40	0.28	0.26
Agricultural land $\ge$ 1.0	0.87	0.49	0.31	0.56
Household income and composition				
Household income	24,571	17,140	23,088	15,604
Crop income	10,655	7584	8618	8684
Sidelines	6536	6100	2285	2512
Wages	4720	2221	5907	3178
Family business	2488	207	4534	328
Other income	−531	593	472	471
Remittances	703	436	1272	431
Per capita income	5166	3140	5239	2808
Simple demographics				
Household size	5.13	5.72	4.67	5.86
Maximum male education	6.70	3.68	8.24	4.47
Maximum female education	5.09	2.52	7.82	3.37
Household labor (days per year)				
Male, Days in farming	204	203	161	212
Male, Days in non-farm work	42	3	46	9
Female, Days in farming	142	243	135	216
Female, Days in non-farm work	46	1	70	10
Main ethnic groups (%)				
Xo Dang (Sedang)		50		
Ba Na (Bahnar)		25		
Gie Trieng		23		
Ngai				32
E De (Rhade)				22
Co Ho				16
*N*	23	207	267	629

*Notes:* (1) Source: VHLSS 2002; (2) Household education is the years of education of the highest educated (male or female) adult in the household. This is calculated for household members 15 and older. If there is no male or female older than 15, the maximum is calculated as 0; (3) Income variables are expressed in ’000 VND (2007 prices); (4) Land Distribution based on households with land by size (in hectares)

We report summary measures relating to both the mean and distribution of landholdings for annual, perennial, and annual plus perennial land for minority and non-minority households. The land indicators in the surveys do not perfectly line up with the categories in the policy documents (annual and perennial land in the survey; terrace vs. paddy land in the policy documents). The policy was directed primarily at annual land, though as land is clearly fungible, and perennials are important in the Central Highlands, it makes sense to explore the sensitivity of conclusions to various definitions of land holdings. It is also unlikely that households with significant holdings of perennial, but not annual land, were the intended beneficiaries of the program.

In the case of Kon Tum, ethnic minority landholdings were significantly smaller than those of their Kinh counterparts. Average total ethnic minority landholdings were 1.12 hectares per household compared to 1.97 for Kinh, or a difference of nearly 75 percent.^6^ Minority households owned less of both types of land, with their holdings of perennial land only 0.06 hectares compared to 0.55 hectares for non-minorities. We also observe 12% of ethnic minority households having annual landholdings less than 0.5 hectares, and an additional 41% with landholdings between 0.5 and 1.0 hectares. In total, 53% of all ethnic minority households have less than a hectare of annual land. Including perennial land only marginally lowers the percentage, reflecting the small amount of perennial land held. Our data suggest that about half of minority households were eligible for Program 132. By comparison, only 13% of non-ethnic minority households have annual plus perennial land less than a hectare. One striking difference between minority and Kinh households is the role played by forestland. Minority households claim access to almost three-quarters of a hectare of forestland, while it is essentially zero for Kinh households.

A slightly different picture emerges outside Kon Tum. Ethnic minority households on average have more land than the Kinh, a product of larger holdings of annual land. Holdings of perennial land are nearly identical. There remains a significant percentage of ethnic minorities with annual or total agricultural land holdings less than either 0.5 or 1.0 hectare, but the percentage is typically no higher, and usually lower than we observe for the Kinh. Overall, 69% of Kinh households report landholdings less than a hectare, compared to 44% for ethnic minority households. As in Kon Tum, about half of the ethnic minority households appear eligible for program participation. Unlike Kon Tum, forestland is relatively unimportant in the rest of the Central Highlands.

In the next part of Table 2, we make similar comparisons with respect to household incomes. The differences between Kon Tum and the three remaining Central Highland provinces are relatively small: Ethnic minority household incomes are roughly 30% lower in both cases. In per capita terms, the differences between provinces are larger, reflecting differences in average household size. The composition of income also features important differences. Outside Kon Tum, income from cropping is nearly identical for the two groups, with higher wage and business income for non-ethnic minorities generating much of the gap. In Kon Tum, on the other hand, differences in cropping income between the two groups are the source of slightly less than half of the difference, with income from wages and family businesses making up the rest. Differences in access to land likely underlie the differences in cropping income. Comparing ethnic minorities across provinces, income levels are similar, so the distinction between Kon Tum and the other provinces (in 2002) is not income-based. Nonetheless, the composition of income is different, with ethnic minorities in Kon Tum earning less from cropping, and more from agricultural sidelines, likely as a result of their greater access to forestland.

Comparing other key variables, ethnic minority households are significantly larger than their Kinh neighbors are, with almost six members per household, vs. five for the Kinh. There are striking differences in levels of education across households. First, households in Kon Tum have about 1.5 years less education per person (measured by the most educated adult in the household), irrespective of ethnicity. The gap between ethnic groups is staggering: Almost 3 to 4 full years of education, with Kinh having almost double the years of schooling. To the extent that human capital is an important determinant of income, on and off the farm, it seems at the outset that improving education for ethnic minorities might yield a bigger bang than changing the land distribution. This is consistent with previous researchers (i.e., Van de Walle and Gunewardena (2001), and Baulch et al. (2007, 2012)), who demonstrate that observed differences in access to land explain very little of the gap between ethnic minority and Kinh incomes. Education, on the other hand, is a major contributor. We also explore time-use patterns across households to gauge how improved access to land might affect employment of potentially underutilized ethnic minority family members. Overall, ethnic minority and Kinh households spend a similar total number of days working, but the Kinh spend significantly more time than ethnic minorities in non-farm activities. There are only minor differences in days worked between Kon Tum and elsewhere in the Central Highlands, and men and women have similar employment patterns.

Finally, we report the three largest ethnic groups in each region. Throughout our paper we discuss ethnic minorities as a homogeneous group, when in fact there are many different ethnic groups. Of particular note, the ethnic groups in Kon Tum are different from those in the rest of the Central Highlands, adding another reason why we separate our discussion for these sub-regions.

## Who received land?

In order to evaluate the impact of the program on household incomes, we need to know how program land was allocated to households. Did household eligibility line up with actual program participation (treatment)? Were there deviations from program design that undermine our use of predicted eligibility as the foundation for an identification strategy? Is there evidence that the program was implemented differently than intended?

We begin with a summary in Table 3 of commune officials’ responses to how Programs 132 and 134 were implemented at the local level. Note that our strategy for selecting the 50 communes implies that these estimates may not perfectly line up with the provincial estimates. Since we selected communes based on potential eligibility, the sign and magnitude of the bias will depend on how treatment rates are correlated with potential eligibility.

**Table 3:** Commune reports of implementation (Programs 132 and 134)

	Basic counts			Implementation of Program 132							
					Land for eligibility (communes)				“Treatment Rates”		
	Number of:				Type of land			Threshold	Percentage of ethnic households receiving land		
Province	Communes	Total households	Ethnic minority households	Participating communes	Annual	Perennial	Unused	(Average, in Ha.)	Program 132	Program 134	Program 132 or 134
Kon Tum	10	9724	6492	10	10	0	0	0.86	37.0	1.1	38.1
Gia Lai	15	16,488	9880	12	7	1	4	0.28	10.4	2.0	12.5
Dak Lak	17	42,374	14,412	12	6	5	5	0.61	7.6	3.7	11.3
Lam Dong	8	11,760	6194	1	0	0	1	0.30	0.9	4.8	5.7
Total	50	80,346	36,978	35	23	6	10	0.56	12.4	3.0	15.4

*Notes:* (1) This table reports results from the commune-level CHVLSS surveys; (2) Land for eligibility refers to the land type used by communes to establish household eligibility for Program 132; (3) The threshold is the reported level of land below which households were deemed eligible for program participation; (4) “Treatment Rates” is the percentage of ethnic minority households receiving land from the relevant program

The first three columns of Table 3 report by province the number of communes and households in our sample. Ethnic minority households comprise a significant majority in the Kon Tum and Gia Lai communes, about one-third of households in Dak Lak, and about half of households in the Lam Dong communes. Out of our 50 communes, 35 reported implementing Program 132.^7^ In the next four columns, we summarize the criteria used by communes for establishing household program eligibility. First, what type of land was considered? Eligibility was typically defined in terms of one type of land, e.g., annual, perennial, or unused, but there were a few communes that based it on a combination of types. Most used annual land to establish eligibility, presumably in line with local standards of land use. In Kon Tum, all communes used annual land. In Dak Lak, by contrast, eligibility was frequently based on having too little perennial land. We also find significant differences in the thresholds that were used. Kon Tum appears to have followed the national directives most carefully, with mean eligibility just slightly less than a hectare of annual land. In both Gia Lai and Lam Dong, households typically with land less than 0.3 hectare were deemed eligible, while in Dak Lak it was two times that. Moreover, in Dak Lak, eligibility was sometimes based on perennial land.

These thresholds varied for several reasons, the most important of which was local land availability. In land abundant areas like Kon Tum, it was easier to find land to top up a household to 1.0 hectare, while in land scarce areas, this was too expensive. Moreover, the implied value of a land transfer would have been higher in these communes. In short, the amount of land transferred and the targeting of land to households is correlated with the value of land itself. As a result, the returns to program participation may be correlated with household eligibility, at least across communes. In land abundant areas, a hectare of land may not have amounted to much benefit, especially if it needed significant reclamation.

In Appendix C Table 1, we draw on the commune level data to calculate the type and source of land redistributed to households. In Kon Tum, it is primarily annual land that has been transferred from state farms or plantation, or been reclaimed. The average amount of land redistributed per household is 0.45 hectares, which is identical to the provincial-based estimate. Outside Kon Tum, annual, and perennial land transferred from state farms and plantations is slightly less important, while land obtained by the state from other households (with compensation) makes up nearly a third. The commune level data also imply that on average 0.30 hectares per household were redistributed, which is slightly lower than the provincial level data suggest outside Kon Tum.

In the final three columns of Table 3, we summarize treatment rates based on commune-level responses. The reported participation, or treatment rates should be compared to the final column of Table 1 (based on province-level reports). In Kon Tum, 37% of minority households received land from Program 132, very close to the provincially reported 37.2%. As the line between Program 132 and 134 may be blurry, we also calculate the treatment rate for Program 134 and for Programs 132 and 134 together. In Kon Tum, this bumps the treatment rate marginally, to 38.1%. In the other provinces, the percentage of minority households receiving land under either program was much smaller. The combined treatment rates in Gia Lai (12.5%) and Dak Lak (11.7%) are still reasonably close to the provincial reports of 15.7% and 10.3%. For Lam Dong, we only had one out of eight communes participating in Program 132 and a few more in Program 134. The resulting treatment rate is very low, at 5.7%, and is significantly below the provincially reported rate of 19.4%. This may be the result of some combination of poor luck of the draw with our sample of communes, and over-reporting by provincial authorities. Regardless, it implies a sample for which it will be difficult to estimate reliably the impact of program participation. Even with treatment rates of 10 percent in Gia Lai and Dak Lak, the number of treated households is very small.

In summary, the province and commune level data reveal significant heterogeneity in treatment rates. This heterogeneity likely comes from a number of sources including: (1) variation in the cut-offs used for determining eligibility; (2) variation in the number of eligible households; and (3) most importantly, variation in available land, and budget for project implementation.

### Household-level measures of program participation

We do not know whether a particular household was deemed eligible for the program. However, as discussed in the context of Table 2, we observe land holdings in 2002, which should be strongly correlated with eligibility. Strictly speaking, our estimates of eligibility are for potential eligibility, and for expositional ease, we will mostly dispense with the “potential” qualifier. Recall also that the VHLSS land data do not map perfectly into the eligibility criteria spelled out in the Program 132 and 134 documents, which were based on terrace land. In Table 4, we tabulate the proportion of households falling into the same four basic landholdings groups: no land, 0 to 0.5 hectares, 0.5 to 1.0 hectares and more than 1.0 hectare. In Kon Tum, 50.7% of ethnic minority households have less than a hectare of land, while 43.7% have less than a hectare elsewhere in the Central Highlands. How many of these households received land from the program?

**Table 4:** Household-level reports of program participation (“Treatment” rates) by 2002 land holdings

		Potentially eligible (based on initial land holdings, (Ha.))				“Ineligible”	F-tests (*p*-values)	
	All	0	0 to 0.5	0.5 to 1.0	< 1.0	≥1.0	OLS	FE
Kon Tum								
Treat 1: 132 Indicator (%)	16.4	0.0	8.7	23.2	20.0	12.7	2.29 (0.16)	2.43 (0.15)
Treat 2: 132 or 134 Indicator (%)	17.4	0.0	8.7	23.2	20.0	14.7	1.77 (0.23)	1.40 (0.30)
Treat 3: 132, 134, or Reclaimed (%)	31.9	0.0	21.7	40.2	36.2	27.5	***4.98 (0.04)***	2.13 (0.18)
Sample size	207	0	23	82	105	102	207	207
Percentage of households	100	0.0	11.1	39.6	50.7	49.3	—	—
Non-Kon Tum								
Treat 1: 132 Indicator (%)	2.1	0.0	0.0	1.2	0.7	3.1	1.18 (0.32)	0.87 (0.47)
Treat 2: 132 or 134 Indicator (%)	3.7	0.0	3.8	4.3	4.0	3.4	2.16 (0.11)	0.86 (0.47)
Treat 3: 132, 134, or Reclaimed (%)	9.9	16.7	7.6	8.5	8.4	11.0	0.41 (0.75)	0.47 (0.70)
Sample size	629	6	105	164	275	354	629	629
Percentage of households	100	1.0	16.7	26.1	43.7	56.3		

*Notes:* (1) Source: VHLSS 2002 and CHVLSS 2008; (2) Treatment status is based on household reports from the CHVLSS 2008; (3) “Treat 1”, “Treat 2”, and “Treat 3” refer to different potential definitions of self-reported treatment status, depending on household responses to questions pertaining to participation. (4) Potential Eligibility is based on reported land holdings in 2002 (combined annual plus perennial land). The column “All” refers to all minority households in the province, and gives the overall “treatment rate.”; (5) All calculations based on sample of minority households only; (6) F-tests test whether there is a correlation between treatment status and being in a “potential eligibility” category. This is based on a regression of treatment status on indicator variables for initial landholdings being in the zero, zero to 0.5, or 0.5 to 1.0 categories. The F-tests are computed with (“FE”), and without (“OLS”) commune dummies; (8) Robust F-statistics calculated with Variance-Covariance clustered at the commune level; (7) Statistically significant F-statistics (at the 5% level), highlighted in bold italics

In our survey, we directly asked households whether they received land from Programs 132 or 134. While program eligibility status is fuzzy for reasons described above, even treatment status is potentially ambiguous, as households may not be fully aware of their own treatment status. We, therefore, provide information on treatment by initial landholdings using several alternative definitions of treatment: (1) whether the household reports receiving land from Program 132; (2) whether the household reports receiving land from Programs 132 or 134; and (3) whether the household reports receiving land from Programs 132 or 134, or report they reclaimed land since 2002. Our third measure of treatment is the most liberal estimate of treatment, and allows for the possibility that households may not know the channel by which they received the additional land. A significant amount of program land required reclaimation, and land reclaimed by households may have been program land, even if they did not remember the legal source.^8^ However, this treatment measure may also include land that has nothing to do with the program.

Turning to the cross tabulations in Table 4, we begin with the overall treatment rates, irrespective of eligibility (the “All” column in Table 4). In Kon Tum, 16.4% of all ethnic minority households received land through Program 132, and marginally higher, 17.4%, if Program 134 land is included. Under our broadest measure of potential treatment, nearly a third of ethnic minority households in Kon Tum received land, which is similar to treatment rates reported at the commune and provincial level. Household-reported treatment rates outside of Kon Tum are significantly lower. Only 2.1% of all ethnic minority households received land from 132, with an additional 1.6%, or 3.7% in total, receiving land from Programs 132 or 134. If we include newly reclaimed land since 2002 that may have also come from the state, 9.9% of all households received land since 2002, which lines up better with the commune and provincial reports. The household data confirm the much higher rates of program participation in Kon Tum, and the low rates of participation outside Kon Tum using the narrowest definition of treatment.^9^

The implied participation rates are still lower than the official sources, especially if we use the measures of treatment that most directly refer to program land. Why might this be the case? There are two possible explanations aside from over-reporting by local governments. First, imperfect recall by households over the legal sources of their land over the previous 6 years may be a factor. While households might be familiar with the programs, attributing an individual plot of land to the program may be difficult. This will be especially true for land that they may have been informally working prior to Program 132, and where the program effectively secured property rights to existing land. It is less likely that program land has already been disposed of, given the restrictions placed on selling this land. Of course, less formal transfers (i.e., to children) could lead to leakage at the household level.

Second, new household formation and our sample design may also be a contributing factor to the gap. An examination of the 2001 and 2006 Agricultural Censuses reveals a significant increase in the number of ethnic minority households, and a decline in average household size over a period that spanned the implementation of Program 132. We do not believe that Program 132 alone caused a significant increase in household formation – the value of the relevant land is not high enough for that – but other factors could coincide with ethnic minority household splitting and formation. The housing component of Program 134, for example, may have encouraged younger generations to form their own households. Formation of new households would have implications for the estimation of official, aggregate participation rates: If land was given to new households, then the appropriate denominator would include the new households, not just the original 2002 households. *Ex post* household treatment rates (treated households divided by the original number of households) would be exaggerated. Furthermore, new households make reconciling results from the panel and the aggregate sample more difficult. By construction, the panel nature of our data limits our ability to measure treatment of new, younger households. Especially if the new households are more likely eligible than the panel households, our sample will understate the extent of treatment, while giving an accurate estimate of treatment in the base households from 2002. This highlights an inevitable limitation of retrospective, panel-based surveys.^10^

As for linkages with eligibility, the easiest comparison is between those households with less than or greater than one hectare of land (i.e., potentially eligible to those who should not in principle receive any land). In Kon Tum, as well as in the remaining Central Highland provinces, there appears to be significant leakage in the treatment, with the ineligible almost equally likely to be treated. In Kon Tum, 20% of households with land in 2002 under one hectare received land from Program 132 or 134, while 14.7% of ineligible households also received program land. While it is true that eligible households had a higher probability of treatment, the relationship between eligibility and treatment seems very weak. In the final two columns of Table 4, we formally test for a relationship between land holdings in 2002 and reported treatment status by regressing treatment status on indicators of a household’s landholding category. We report the F-statistic for the land category dummies. We do this with, and without commune dummies, allowing for some heterogeneity of average farm sizes and land availability across communes. For Kon Tum, neither of our first two measures of treatment are statistically significantly correlated with 2002 landholding status. If we include reclaimed land in the measure of treatment, there is a stronger link, but this disappears once we account for commune-level heterogeneity. Outside Kon Tum, the leakage appears to have been just as severe. Using the broadest measure of treatment, slightly more ineligible households received land (11.0% vs. 8.4%). Stated differently, between one-half to two-thirds of treated households were ineligible based on 2002 land holdings, depending on the treatment measure used. There is no significant link between predicted eligibility and treatment status.^11^

### How progressive were the land transfers?

An underlying premise of Programs 132 and 134, and the allocation of land to ethnic minorities is the view that landholdings are positively correlated with household incomes. Thus, targeting land for land-poor households will improve the welfare of the neediest rural ethnic minority households. Land transferred through 132/134 may not have been directed to the land poor, but it may still have been directed to poor households. In Table 5, we provide a breakdown of landholdings and treatment by 2002 per capita income quartiles for Kon Tum, and then the rest of the Central Highlands. In Kon Tum, there is only a weak relationship between average landholdings and income quartile. Largely because of differences in annual land, average landholdings fall marginally between the first and second quartiles, and then rise slightly through the fourth. Outside Kon Tum, the link is much stronger, especially with respect to perennial land, which rises from 0.25 hectares in the first quartile to 0.71 in the fourth. In total, households in the richest quartile have 75 percent more agricultural land than households in the poorest quartile do. In general, annual land is only weakly related to household income, and is a poor marker of low income. Perennial holdings, on the other hand, are more concentrated among richer households. It takes money to invest in this type of land, from the preparation of the land itself, the cost of the planting and maintaining the perennials, and the foregone income while waiting for the plants to mature.

**Table 5:** 2002 Land status and 2008 reported treatment rates by 2002 income quartile

	Means by income quartile				F-tests (*p*-values)	
	1	2	3	4	OLS	FE
Kon Tum:						
Per capita household income (2002)	1712	2148	3112	4307	***213 (0.00)***	***120 (0.00)***
Annual land in 2002 (Ha.)	1.01	0.89	1.07	1.17	3.07 (0.09)	1.36 (0.32)
Perennial land in 2002 (Ha.)	0.05	0.09	0.06	0.05	0.29 (0.83)	0.67 (0.60)
Total agricultural land in 2002 (Ha.)	1.06	0.99	1.14	1.22	1.47 (0.29)	0.62 (0.62)
Potentially eligible (total land < 1.0 Ha.)	0.56	0.73	0.45	0.40	5.48 (0.02)	2.24 (0.08)
Treat 1: 132 Indicator (%)	11.1	20.5	25.0	8.3	***6.17 (0.02)***	2.03 (0.19)
Treat 2: 132 or 134 Indicator (%)	11.1	20.5	26.6	9.7	***6.50 (0.02)***	2.90 (0.10)
Treat 3: 132, 134, or Reclaimed (%)	18.5	34.1	46.9	22.2	2.62 (0.12)	1.85 (0.22)
Non-Kon Tum Central Highlands:						
Per capita household income (2002)	1400	2278	2938	5178	***506 (0.00)***	***287 (0.00)***
Annual land in 2002 (Ha.)	0.70	0.77	0.66	0.97	1.87 (0.15)	***3.70 (0.02)***
Perennial land in 2002 (Ha.)	0.25	0.32	0.67	0.71	***6.88 (0.00)***	***10.47 (0.00)***
Total agricultural land in 2002 (Ha.)	0.95	1.09	1.33	1.68	***8.70 (0.00)***	***12.00 (0.00)***
Potentially eligible (Total land < 1.0 Ha.)	0.54	0.47	0.40	0.30	***5.72 (0.00)***	***10.12 (0.00)***
Treat 1: 132 Indicator (%)	2.2	0.0	4.8	1.5	1.05 (0.38)	0.95 (0.43)
Treat 2: 132 or 134 Indicator (%)	3.8	3.0	6.2	1.5	0.89 (0.46)	0.26 (0.85)
Treat 3: 132, 134, or Reclaimed (%)	10.4	10.9	11.0	6.6	0.67 (0.58)	0.29 (0.83)

*Notes:* (1) This table reports F-statistics for the joint significance of household variables of a given type, in a regression explaining household reported treatment status; (2) Household characteristics include: log per capita household income in 2002, household size in 2002, and maximum male and female education in 2002; (3) The F-tests are computed with (“FE”), and without (“OLS”) commune dummies; (4) Robust F-statistics calculated with Variance-Covariance clustered at the commune level; (5) Statistically significant F-statistics (at the 5% level), highlighted in bold italics

In Table 5, we also report potential eligibility rates by income quartile group, based on whether a household had less than one hectare of land in 2002. Eligibility typically falls through the quartiles, but the drop is much less than might be expected. In Kon Tum, the first quartile (Q1) has 56% of households with agricultural land under one hectare. This is actually lower than the next richest quartile (Q2), where 73% are potentially eligible. Eligibility rates then fall through the next two quartiles. Outside Kon Tum, the link between potential eligibility and income quartile is stronger. This reflects the greater importance of more lucrative perennial land where there is a stronger link between acreage and income (as opposed to some of the larger more marginal farms of annual land in Kon Tum).

Of most interest from an equity perspective, household-reported treatment rates are fairly similar across quartiles, though higher in the middle. In Kon Tum, treatment rates peak in the third quartile. The same pattern holds outside Kon Tum, though the treatment rates are much lower in magnitude. In summary, land transfers were not disproportionately directed to land poor households. Nor were transfers directed toward poorer households. If anything, transfers went to the middle and upper income households, contrary to the stated objectives from Hanoi.

As we ultimately wish to relate treatment to outcomes, was land targeted to certain types of households? To explore the issue further, we estimate richer versions of the treatment regressions reported in Table 4. In Table 6 we show the results of regressions relating Treat 2 (received land from either 132 or 134), our preferred measure of program participation, to a more detailed set of land holding indicators (i.e., separate indicators for the amount of annual, perennial, and forest land), as well as household characteristics (per capita income, education, and family size). As in Table 4, we report F-statistics for the significance of the regressors in predicting treatment. The bottom line from Table 6 is that we find very little that is correlated with treatment status, except which commune a household resides. Based on 2002 observables, it is difficult to predict who would receive program land.

**Table 6:** Exploring linkages between potential eligibility, other household characteristics, and treatment status (based on Treat 2)

	Kon Tum Central Highlands		Non-Kon Tum	
	OLS	FE	OLS	FE
F-Annual land categories	2.423	1.488	0.187	0.634
	(0.150)	(0.282)	(0.905)	(0.599)
F Perennial land categories	***5.254***	***4.518***	0.971	1.104
	***(0.027)***	***(0.039)***	(0.418)	(0.361)
F Forestry land categories	0.058	1.666	***8.130***	***5.247***
	(0.944)	(0.248)	***(0.001)***	***(0.011)***
F ALL land categories	***8.015***	***14.604***	***4.151***	2.104
	***(0.004)***	***(0.001)***	***(0.002)***	(0.064)
F Household characteristics	0.212	0.896	1.700	1.542
	(0.925)	(0.509)	(0.173)	(0.213)

*Notes:* (1) This table reports F-statistics for the joint significance of household variables of a given type, in a regression explaining household reported treatment status as measured by “Treat 2” (whether the program reports participation in either Program 132 or 134); (2) Household characteristics include: log per capita household income in 2002, household size in 2002, and maximum male and female education in 2002; (3) The F-tests are computed with (“FE”), and without (“OLS”) commune dummies; (4) Robust F-statistics calculated with Variance-Covariance clustered at the commune level; (5) Statistically significant F-statistics (at the 5% level), highlighted in bold italics

In Kon Tum, we find that households with less perennial land were slightly more likely to receive land. Outside Kon Tum, we find no evidence that households with less farm land were more likely to be treated. On the other hand, while forestland holdings are rare at the household-level outside Kon Tum, there is some correlation between having forestland, and treatment status. None of the household characteristics, notably income and education, were correlated with treatment. The most significant variables, by far, are the commune dummies.

There are several implications of this exercise. First, there is very little predictable variation of treatment within communes. The program was not implemented the way that it was designed, at least based on the three-page sketch from Hanoi. The variation of treatment across communes means that it is not likely to be valid to exclude commune dummies from any analysis. The most reliable analysis will have to be conducted within communes. The nature of the implementation of the program also underscores the value of separating Kon Tum from the rest of the analysis. The absence of a link between observable household characteristics in 2002, especially land holdings, and subsequent treatment, undermines any identification strategy that relies on these serving as instruments, either for conventional Instrumental Variables, or for Fuzzy Regression Discontinuity. However, there is no evidence of departures from random assignment, at least to the extent that program participation excluded those with the highest income or education. We still need to be concerned that households were granted land based on the perceived value of land to them: Which households could put the land to its best use? At least for Kon Tum, our evidence is also consistent with broad participation of ethnic minority households in the program, with land assigned essentially randomly to households (at least, based on observables). Furthermore, as emphasized by Alatas et al. (2012), and Karlan and Thuysbaert (2016), local satisfaction may be higher with such deviations from the strict targeting guidelines, as long as the actual redistribution is perceived as “fair.” We now turn to an analysis of what happened to the observable outcomes of these households after the implementation of Program 132.

## Assessing the impact of Programs 132 and 134

We are interested in assessing the impact that the distribution of land through Programs 132 and 134 had on rural household incomes in the Central Highlands between 2002 and 2008. Given that our identification strategy will be driven by before-and-after comparisons between treated and untreated households, it is useful to look at the broader changes that occurred in both land and incomes over this 6-year period.

### Changes in land

We wish to know whether minority households, as a group, experienced improvements in their land holdings. We have detailed information in the 2002 and 2008 surveys to compare household land holdings irrespective of treatment status, and we also asked households in 2008 whether they received land from the programs. We can compare changes in the land distribution between minorities and Kinh—who would not have been treated—and also between ethnic minority households that report being treated or not.

In Table 7, we report land outcomes for ethnic minority and Kinh households in the Central Highlands. For ethnic minority households, the data suggest an increase in average agricultural land of slightly more than 20 percent, from 1.20 to 1.47 hectares per household. This was offset by the loss of almost all of the forestland households reported having in 2002.^12^ By comparison, agricultural landholdings increased from 0.89 to 0.97 hectares for the Kinh. Overall, the data suggest that there was a relative increase in land holdings for minorities, consistent with the existence of the programs. Separate breakdowns for Kon Tum and non-Kon Tum are once again helpful. Ethnic minority households in both parts of the Central Highlands experienced an increase in landholdings of agricultural land, with households outside Kon Tum reporting a slightly larger increase, from 1.23 to 1.52 hectares. All of the reduction in forestry land occurs in Kon Tum.

**Table 7:** Land outcomes: 2002 vs. 2008

	All Central Highlands				Ethnic minority only			
	Kinh		Minority		Kon Tum		Non-Kon Tum	
	2002	2008	2002	2008	2002	2008	2002	2008
Average annual land (Ha.)	0.40	0.30	0.84	1.02	1.06	1.14	0.77	0.98
Average perennial land (Ha.)	0.49	0.67	0.37	0.45	0.06	0.17	0.47	0.54
Average agricultural land (Ha.)	0.89	0.97	1.20	1.47	1.12	1.31	1.23	1.52
Average forestry land (Ha.)	0.02	0.01	0.21	0.03	0.76	0.00	0.03	0.03
Percentage of households with land:								
Equal to 0 Ha.	9.0	18.6	0.7	2.3	0.0	0.5	1.0	2.9
Less than 0.5 Ha.	38.3	37.2	16.0	15.6	11.1	15.0	17.6	15.7
Less than 1.0 Ha.	64.5	60.3	45.5	38.9	50.7	43.5	43.7	37.4

*Notes:* (1) Source: VHLSS 2002 and CHVLSS 2008; (2) Land variables are expressed as average hectares per household; (3) The percentages of household with land below a particular cutoff is also the “Cumulative Distribution Function,” (or CDF) of the land distribution

The shares of households by land-size category (cumulative distribution functions, or CDF’s, of land) reveal that the increase in mean landholdings was accompanied by similar reductions in the percentage of households with less than a hectare of agricultural land. In Kon Tum, the percentage of households with land under one hectare declines from 50.7% to 43.5%. Outside Kon Tum, there is a similar sized drop in the percentage of households with less than a hectare of farmland, from 43.7% to 37.4%. Note that for the Kinh the percentage of households with zero land doubles, from 9.0% to 18.6%. Even while some Kinh households were expanding their plantings of perennials, other Kinh households were exiting agriculture. This also corroborates the point raised in Ravallion and van de Walle (2008) that landlessness is a poor proxy for risk of poverty, and is frequently a sign that households are moving out of agriculture into pursuits that are more lucrative. This highlights the challenge of using more or less land as a measure of household improvement.

**Fig. 1:**
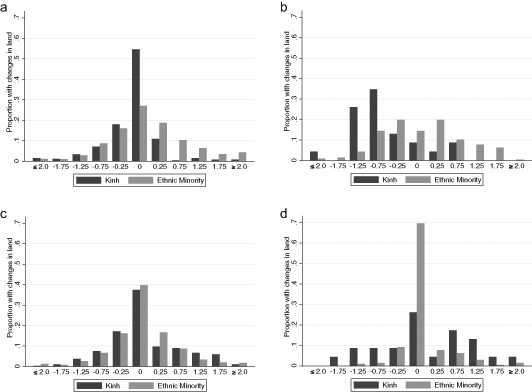
Changes of household land holdings between 2002 and 2008: what proportion of households had changes of “X” hectares? **a** Annual land, outside Kon Tum, **b** Annual Land, Kon Tum, **c** Perennial land, outside Kon Tum, and **d** Perennial land, Kon Tum

These simple summaries of the aggregate land distribution hide important subtleties. First, in moving from annual to perennial land, farm sizes generally shrink (so overall land is a poor summary of farm capacity). Second, there is a great deal of shuffling between annual and perennial land that may have an even greater impact on income changes than the simple total acreage. Finally, the averages hide the significant amount of churning in land among households. In Fig. 1, we plot histograms of changes in land holdings for the Kinh and minority households. The change in land holdings is simply the difference between 2008 and 2002 household cultivated land, calculated separately for annual and perennial land. To allow for some measurement error, we allow small changes to count as zero change. We then calculate the fraction of households with increases or decreases of land of varying amounts. In addition to showing the underlying heterogeneity of changes, and movements into annual or perennial land, we may also be able to detect program participation indirectly: Do we observe significant numbers of ethnic minority households receiving land between zero and one hectare?

Starting with Kon Tum, the vast majority of households experienced changes in annual land holdings of at least 0.25 hectares. For ethnic minority households, annual land increased upwards from 0.25 hectares, consistent with having participated in Program 132. Some households also experienced declines in their land holdings. Kinh households divested themselves of annual land. For perennial land, changes in both directions largely applied to the Kinh, with increases outweighing decreases. Outside Kon Tum, we also witness a majority of ethnic minority households experiencing changes in their annual land holdings, with increases of at least 0.25 hectares. Compared to Kon Tum, ethnic minority households elsewhere in the Central Highlands shifted into perennial land. Except for perennial land in Kon Tum, the panels of Fig. 1 show that a significant share of ethnic minority households improved their land holdings relative to their Kinh neighbors.

What does this imply about potential linkages between Program 132 and the land distribution? One possibility is that these changes are largely a consequence of the policy. In our survey, we asked whether households had acquired new land since 2002. Overall, more than a quarter of all ethnic minority households reported new land that they had acquired since 2002, with the percentage in Kon Tum 44.2%. In Kon Tum, the three primary sources of this land are identified as coming through Programs 132 or 134, newly reclaimed land, and land that they were allocated long-term use rights to (presumably by the Commune). Outside Kon Tum, 24.0% of all households report acquiring new land, but land that might be linked to the state is significantly less than in Kon Tum. More important is land that is either inherited, or bought. Clearly, program land is not the only way for ethnic minorities to have increased their land holdings between 2002 and 2008. This complicates the estimation of treatment effects, as land of the untreated households changed through other means than the program. The existence of other channels to acquire land makes it more difficult to distinguish differential outcomes for treated and untreated households, especially outside Kon Tum.^13^

### Changes in income

In Table 8, we report a breakdown of real household incomes and expenditures for 2002 and 2008, all expressed in constant 2008 VND, and data on household labor supply. We present results for Kinh and minority households over the entire Central Highlands, as well as a separate breakdown for minority households inside and outside Kon Tum. We focus our discussion on minority households, but also review the dramatic change in outcomes for Kinh households. Household incomes more than doubled in real terms, from 23.2 million VND to 48.8 million VND. While incomes rose significantly from most sources, the dramatic increase in crop income drove the overall increase. This reflected the combined effect of higher output from perennial land and higher prices for perennial crops. Despite the role of agriculture in the growth of income, Kinh households devoted less labor time to farming, and more time to non-agricultural pursuits in 2008 compared to 2002.

**Table 8:** Changes to income and work, 2002–2008 (panel households)

	All Central Highlands				Minority only			
	Kinh		Minority		Kon Tum		Non-Kon Tum	
	2002	2008	2002	2008	2002	2008	2002	2008
Income								
Per capita income	5233	11,666	2890	4906	3140	2921	2808	5559
Per capita expenditure	4370	7574	2227	3250	2517	2304	2165	3562
Log per capita income	8.40	9.13	7.85	8.24	7.99	7.86	7.81	8.36
Log per capita expenditure	8.27	8.81	7.62	7.96	7.78	7.67	7.59	8.05
Household income	23,206	48,805	15,984	26,175	17,140	15,837	15,604	29,577
Crop income	8780	27,791	8412	17,270	7584	9686	8684	19,766
Sidelines	2623	2072	3400	2108	6100	1287	2512	2378
Wages	5812	8640	2941	4548	2221	3169	3178	5002
Family business	4371	7225	298	314	207	185	328	356
Other Income	392	1283	501	1030	593	1144	471	992
Remittances	1227	1794	432	905	436	366	431	1083
Labor (days worked)								
Male, days in farming	164	140	210	226	203	245	212	219
Male, days in non-farm work	46	65	7	13	3	8	9	15
Female, days in farming	135	116	223	219	243	258	216	206
Female, days in non-farm work	68	62	8	10	1	3	10	12

*Notes:* (1) Source: VHLSS 2002 and CHVLSS 2008 (Panel Households); (2) All values are expressed in constant ’000 VND (2007 prices)

Turning to ethnic minority households, in 2002 real household incomes in Kon Tum were 17.1 million VND. Per capita incomes were 3.1 million, while per capita expenditures were slightly lower at 2.5, implying that these households were saving in upwards of twenty percent of their incomes. Nearly three-quarters of income came from cropping and agriculture-related sidelines, e.g., forestry and animal husbandry, with income from wages making up most of the rest. Incomes outside Kon Tum were modestly lower, with income from wages (sidelines) playing a more (less) important role. We observe a sharp divergence in the trends in incomes between provinces. In Kon Tum, total household as well as household per capita real incomes fell between 2002 and 2008. Similar behavior is seen for per capita expenditure, confirming that this is not an artifact of problems in measuring income. Cropping income rose by nearly 30%, and wages by 43%, but these increases are more than offset by the collapse in sideline income, primarily forestry and livestock, of 79%. There are a number of alternative explanations for this decline. The fact that the reduction in sideline incomes included significant losses in earnings from forestry, livestock, and hunting and trapping suggests changing rules on access and use in Kon Tum. In Appendix D we explore the drop in sideline income in more depth. We confirm that the decline is strongly associated with the loss of access to forest land, though the timing of the decline suggests that it was specific to 2008, and had little to do with Program 132 or 134.

Contrast this behavior with the growth in the Central Highlands outside Kon Tum, where per capita incomes almost doubled from 2.8 million to 5.6 million VND. Largely driving this growth is the sky-rocketing growth of income from the cropping sector, which can be linked to land in perennials in 2002. This increase is complemented by growth in wage income, which is slightly larger in percentage terms than we observe in Kon Tum. The Central Highland provinces outside Kon Tum also experience a reduction in incomes from sidelines, but of much smaller magnitude.^14^

### Program evaluation

The aggregate evidence suggests that the changes in the distribution of land are consistent with the implementation of Programs 132 and 134. However, it is not clear whether this is due to the program or part of the dynamic changes in landholdings taking place. Even less obvious is whether program participation did anything to improve ethnic minority living standards. Ethnic minorities outside Kon Tum experienced increases of income far beyond anything a half-hectare of land could generate, while households in Kon Tum saw their incomes decline. In order to better identify the potentially causal links between the Program 132 and household outcomes, we now turn to household-level treatment status and outcomes.

We estimate a program evaluation regression of the form:

$$y_{h,08} - y_{h,02} = \alpha + \lambda y_{h,02} + \beta Treat_{h,08} + \gamma \prime X_{h,02} + u_h$$

where $y_{h,08} - y_{h,02}$ represents the change between 2002 and 2008 for a particular outcome $y_h$ for household $h$; $y_{h,02}$ is the 2002 level of outcome $y_h$; $Treat_{h,08}$ is household reported treatment status from the 2008 survey; and $X_{h,02}$ is a vector of household controls, all dated 2002. We focus on the key outcomes that were the objective of the policy: land and income. To better understand the potential impact of the program, we examine annual and perennial land separately, and also the various sub-components of income. Crop income is of most obvious importance, but given the sharp decline in sideline income, we also examine income from sidelines. One concern is that crop income increased at the expense of sideline income. This could happen if minority households were diverted (or forced) from activities in the forest, in favor of more sedentary agriculture. We also explore labor supply to agriculture to see if access to land affected household time allocation. We might observe greater agricultural activity in terms of labor input, but given production lags with perennials, not yet find an effect of the program on income. As for measuring treatment status, we tried a variety of measures. We report our *ex ante* preferred indicator: Self-reported household participation in Programs 132 or 134 (“Treat 2”). Results are generally not sensitive to this choice.^15^

The standard problem in program evaluation is that the effect of treatment is confounded with unobservable determinants of the outcomes, in this case, changes in land holdings or growth of incomes. The most feasible way to deal with this is to include a rich set of covariates, and assume that treatment status is conditionally independent of the potential outcomes. We include a vector of land holding indicators for the amount of annual, perennial, and forest land controlled in 2002. These variables control for program eligibility, and given the importance of land holdings for income growth, also control for linkages between household land investments prior to 2002 and subsequent income growth. The treatment variable will then identify the effect of new land that potentially came through the program. We exploit the panel structure of the data and also include controls for household predictors of income growth, specifically household education and size in 2002, as well as a vector of household outcomes in 2002, most notably total income (and log per capita income), and income from various sources (crop income, sideline income). To the extent that these variables are correlated with both program participation and potential outcomes, their inclusion helps control for some types of endogenous assignment to the program. Finally, in all specifications, we include commune fixed effects. This allows for the fact that programs were implemented differently across communes, and that these differences are possibly correlated with the potential changes in outcomes across communes. This implies that our specification is identifying the effect of program participation by comparing treated and untreated households within the same commune, holding constant (in a linear regression sense) a household’s land endowments and income in 2002.

The identification strategy rests on the assumption that within communes, otherwise identical households did not receive program land for reasons correlated with unobserved determinants of future income growth, or their ability to access land. What could undermine this? On one side, commune officials might want to give land to those households that could put it to the most productive use. In this case, we expect an upward bias in the returns to program participation. We cannot rule this out, but the fact that we find no correlation between treatment status and other predictors of potential income growth, such as education or income in 2002, leads us to believe that this source of bias is likely small. In the other direction, officials may favor households that most need the land, in which case there is a downward (negative) bias in the estimated coefficients. Since we find no evidence that program participation was higher for low-income households or those with less land, we do not view this as being a large bias.

Our identification strategy uses ethnic minority households in the same commune that did not receive land as the counterfactual. Are there alternative estimation strategies? Based on the wording of Program 132, initially the most promising was to use landholdings from 2002 to construct instruments. As we saw previously, however, land-holdings in 2002 are uncorrelated with reported treatment status, eliminating this as a useful source of instruments. It also turns out that land holdings in 2002 fail to satisfy exclusion restrictions as incomes rose fastest for those households with greater land holdings of perennial land in 2002, and changes in land between 2008 and 2002 were also correlated with land holdings in 2002 for reasons that have nothing to do with program participation. Hence, an instrumental variable strategy based on initial land holdings will not work.

Are there other ways to estimate the counterfactual? The Kinh are not a plausible control group. While not eligible or treated, their income growth is a poor counterfactual for what would happen to minorities in the absence of treatment. In the same vein, minorities outside the Central Highlands are a poor control group, given the heterogeneity of income growth across locations. Moreover, as we cannot reliably disentangle the impact of land received from Program 132 or 134, these minority households cannot serve as a control group as they were treated in Program 134. In summary, and acknowledging its limitations, the best counterfactual for treated minority households in the Central Highlands are untreated minority households in the same commune with similar covariates.

### The value of land: what is the potential impact of treatment?

Before estimating the impact of program participation, we estimate the potential impact that an additional plot of land can have on household income. In Table 9, we report the results of value of land regressions. These regressions allow us to estimate the crop income associated with an additional hectare of annual or perennial land, and to compare these returns by ethnic minority status, or by location. We do this several different ways. First, we estimate cross-section regressions separately for 2002 and 2008, relating crop income from a given year to the land used that year. Columns 1 and 2 report estimates of a regression of the total net income earned by the household from cropping on household holdings of annual and perennial land. Other controls include household size, male and female education, and commune fixed effects. We estimate this specification separately for four samples: (1) All Central Highlands; (2) All ethnic minorities; (3) Ethnic minorities in Kon Tum; and (4) Ethnic minorities outside Kon Tum. In general, we expect the returns to perennial land to exceed that to annual, but recall that land types may only imperfectly capture land use. Moreover, at any given point in time, households may be in the process of shifting some of their annual land into perennial crops. If reclassification of land occurs only with a lag, this could bias the comparison of returns. In addition, it typically takes three to 4 years before newly planted land in perennials begins to generate income.

**Table 9:** How much crop income can be derived from a hectare of land?

	OLS regressions: various specifications			
	(1)	(2)	(3)	(4)
Output measure:	2002 Level	2008 Level	Change	Change
Land measure:	2002 Level	2008 Level	2002 Level	Change
Full sample				
Annual land (Ha.)	***3026***	***5517***	103	***4089***
	***(1076)***	***(1591)***	(1316)	***(1623)***
Perennial land (Ha.)	***5968***	***18,554***	***7608***	***9535***
	***(902)***	***(3579)***	***(3238)***	***(2831)***
Minorities only				
Annual land (Ha.)	***2275***	***4190***	1201	2455
	***(993)***	***(1033)***	(1032)	(1239)
Perennial land (Ha.)	***6103***	***11,622***	671	***7383***
	***(982)***	***(3124)***	(1806)	***(3565)***
Minorities in Kon Tum				
Annual land (Ha.)	***4249***	***4234***	***−2498***	***4480***
	***(772)***	***(685)***	***(801)***	***(660)***
Perennial land (Ha.)	1738	2701	−1728	***3311***
	(1706)	(599)	(9201)	***(951)***
Minorities outside Kon Tum				
Annual land (Ha.)	1026	***4086***	1944	2112
	(1973)	***(1254)***	(1051)	(1383)
Perennial land (Ha.)	***6183***	***13,096***	1009	8106
	***(1022)***	***(3534)***	(1698)	(4182)

*Notes:* (1) Source: VHLSS 2002 and CHVLSS 2008 (Panel); (2) Each column represents a regression of crop income on land holdings (annual and perennial); (3) For crop income: specifications include the 2002 level, 2008 level, and the change between 2008 and 2002; (4) Land holdings are measured as either: the 2002 level, the 2008 level, or the change between 2002 and 2008; (5) All specifications include controls for commune fixed effects, household size, and education; (6) Robust standard errors in parentheses, cluster-corrected at the commune-level, and statistically significant coefficients (5%) in bold italics; (7) The regressions are estimated separately for each subsample

In 2002, for the pooled sample, the returns to perennial land are about double that for annual land. The average return is 5.968 million VND compared to 3.026 for annual land. To help put these numbers in perspective, average per capita incomes in 2002 were approximately 3 million VND. For ethnic minorities in Kon Tum, returns to annual land are higher than for perennial land (4.249 million VND vs. 1.738 million VND), while the reverse is true for ethnic minorities outside Kon Tum, where perennials had much higher returns.^16^

For 2008, the returns to both annual and perennial land are significantly larger, but the increase is much more pronounced in the case of perennials. Amongst the ethnic minority, the returns to both types of land are also higher in 2008, especially for perennials outside Kon Tum. The returns to perennial land could increase for a variety of reasons. The trees (e.g., cashews or coffee) may be more mature and yielding more output, or the prices of these crops may have increased. In fact, it was likely both factors.

These regressions are complemented by two specifications based on changes in crop income between 2002 and 2008. The first (in column 3) is slightly unconventional as a production function. We estimate the change in income as a function of the levels of variables in 2002. This, however, mimics our main program evaluation regressions, where we estimate the effect of program participation conditional on a similar vector of 2002 characteristics. In column 3, we see that those households that had perennial holdings in 2002 had the greatest increases in crop income. Indeed, holding one hectare of annual land (all else equal) did not yield any increase in crop income (though it of course yielded the same level of income per hectare). In this specification, it is clear that access to perennial land in 2002 was critical to increased crop income over this period as being in the annual business yielded no income growth. It also illustrates that perennial holdings in 2002 would be a poor instrument for program participation, as they had a direct effect on crop income growth.

In the last column (4), we estimate a more conventional first difference specification: Were changes in land associated with changes in crop income? This gives us the best possible estimate for the potential impact of program land. For minorities in Kon Tum, we see that an extra hectare of annual land for an ethnic minority household yielded an average of 4.480 million VND, while an extra hectare of perennial land yielded 3.311 million VND. The return to annual land was significantly higher in Kon Tum than outside. As we move to our more formal estimates of the effect of treatment, it is clear, however, that land is a valuable asset for households in the Central Highlands, especially in Kon Tum. Receiving free annual land should increase crop income. An important caveat, however, is that a hectare of program land may not be the same as land acquired in other ways: Households with no experience in cultivating fixed plots of annual land may not be able to achieve the returns suggested by Table 9.^17^

### Program evaluation: results

Regression results for the program evaluation equations are presented in Table 10. Statistically significant results are highlighted, and what is most remarkable is how small the estimated effects are. It is difficult to declare that the program had a large effect on household outcomes, even in Kon Tum where the program was most widespread. However, the results are certainly suggestive that the program had a modest impact.

**Table 10:** Estimated effect of program participation on various outcomes

	Annual land (Ha.)	Perennial land (Ha.)	Total land (Ha.)	Household income (VND)	Crop income (VND)	Sideline income (VND)	Male days farming (days)	Female days farming (days)	Total days farming (days)
Effect for Kon Tum	0.28	−0.04	0.24	***2954***	***2690***	−72	21.1	49.6	70.7
	(0.18)	(0.14)	(0.18)	***(894)***	***(941)***	(177)	(20.3)	(31.4)	(39.9)
Effect for CH outside Kon Tum	−0.3	0.07	−0.23	−4615	−1873	***−970***	16.3	−27.3	−11.0
	(0.16)	(0.18)	(0.21)	(2424)	(2148)	***(432)***	(43.2)	(37.0)	(76.7)

*Notes:* (1) Source: VHLSS 2002 and CHVLSS 2008 (Panel), Ethnic minority Households Only; (2) Each reported coefficient is the regression coefficient on a measure of treatment status from a regression of a change in household outcomes on treatment status (Based on “Treat 2”), and covariates; (3) All specifications include commune fixed effects, flexible controls for household land endowments in 2002, household size and education in 2002, log per capita income in 2002, and a complete set of initial measures of the 2002 dependent variables in this Table; (4) Robust standard errors in parentheses (Cluster-corrected at the commune-level), and statistically significant coefficients (5%) in bold italic

For Kon Tum, we estimate that program households received 0.28 hectare of annual land, which is not far from the official reports, while their holdings of perennial land remain unchanged, again consistent with the official reports. Treated households saw their crop income rise by 2.69 million VND, which is in line (given sampling error) with what 0.28 hectares of additional annual land would generate (from Table 9). Sideline income was unaffected by program participation. This reflects the fact that both treated and untreated households saw their sideline income collapse. Households that participated in the program were not disproportionately diverted out of the forests. In terms of labor supply, both men and women in treated households increased their time in farming, substantially but not statistically significantly. Taken together, we take this as evidence that in Kon Tum, Program 132 provided slightly more annual land to minority households than they would otherwise have been able to obtain, and that they earned income from this land commensurate with the returns to annual land.

In the second row of the table, we report the results for the Central Highlands outside Kon Tum. If anything, we see a negative effect of participation on land accumulation and income. Treated households experienced reductions of annual land, modest increases of perennial land, and reductions of total income, especially sideline income. Very few of the coefficients are statistically significant. To gain a clearer perspective on what may underly the results outside Kon Tum, in Appendix B Table 10 we report results for each province separately. As noted earlier, observed program participation rates are very low, and so results need to be treated with care. We find that Lam Dong has results similar, though less precise than Kon Tum. For Lam Dong, we also find that having forest land in 2002 is a strong predictor of treatment status: For these households, it is plausible that their forest land was taken away, and replaced with annual land. As a result, treated households experienced a decline in sideline income (which likely drives the results in Table 10), but have increased holdings of both annual and perennial land, as well as income from farming. They also spend more time working on the farm. Offsetting the results from Lam Dong, in Dak Lak we observe the opposite phenomenon: Treated households now have *less* annual land, have correspondingly less crop income, and work fewer days in farming. These results are internally consistent, and point to treated households moving disproportionately out of agriculture. It is not obvious why this should be the case. However, as in Lam Dong, having forest land in 2002 is a strong predictor of treatment status in Dak Lak. If treated households lost access to their forest-based farm land, replacement with annual land closer to the village may have left them worse off, with less land and less ability to earn a living from it.

## Discussion and conclusions

At the outset of our project, we expected a relatively clean evaluation of Program 132. We surveyed over 800 minority households yielding a panel of households observed just before, and shortly after the implementation of the program. Official reports suggested that the program was implemented in line with its objectives, transferring land to eligible ethnic minority households with small landholdings. Given the reported levels of participation—60% of eligible households—and compliance with the targeting criteria, we anticipated our main problem would be fine tuning estimates to address potentially endogenous assignment of land to households. As our household survey revealed, however, there is little evidence that the program was implemented in line with official reports. Participation rates were lower than expected, especially outside Kon Tum. Furthermore, there was almost no link between measures of eligibility, initial land holdings, and subsequent treatment.

Our results highlight the value of retrospective surveys in evaluating and auditing the implementation of government programs, and by implication, the value of continuously conducting household surveys that can serve as baselines. With decentralized programs like 132 and 134, it should come as no surprise that local implementation may deviate from the original plan, especially in the case of a land program that hinged on the availability of local land. To predict the impact of this program, a pilot of the program in a sample of randomly selected communes was needed in order to study how local officials interpret and comply with program parameters. As in Alatas et al. (2012), randomization is required at the village-level, not the household level, given the variation of implementation. The lessons from such an exercise could be applied to the design of the policy to take better account of local incentives and constraints for program implementation.

Of course, a more intensive household-level RCT can help evaluate how valuable a plot of land is to a household. On that front, we believe that our more conventional panel-data based estimates are informative. We estimate that households receiving program land experienced increases of income in line with the returns from land distributed through the program. Those returns, while non-negligible, are not large enough to make much difference to the lives of ethnic minority households, and underscore the limitations of land redistribution as a means of raising farm incomes in a dynamic agricultural economy. Households may be able to grow basic staples for household consumption, but serious income growth requires households being able to grow perennials and other crops that require human capital and credit, as well as integration with outside markets. Programs 132 and 134 succeeded in distributing land to a significant number of households, and provided the government with an opportunity to demonstrate its concern over land and ethnic minority issues. Nevertheless, the benefits of the program were diffuse, and far greater investments will be required for ethnic minorities to close appreciably the gap in living standards with their Kinh neighbors.

## Electronic supplementary material

Supplementary Information

## Footnotes

1 See, for example, Van de Walle and Gunewardena 2001, Baulch et al. (2007), Baulch et al. (2010), Baulch, Hung, and Reilly (2012), Dang (2012), and World Bank (2009, 2013) for a more extensive discussion.

2 The Central Highlands provinces include Kon Tum, Gia Lai, Lam Dong, and Dak Lak, which split into Dak Lak and Dak Nong provinces in 2004.

3 Several excellent sources exist. World Bank (2009) provides a broad overview of minority outcomes, history, and policies directed towards improving minority welfare. Writenet (2006) and USAID (2008) provide rich detail on the sources and potential consequences of ethnic conflict. As noted by World Bank (2009) and Dang (2012), while the government has instituted a variety of programs like 132 to address the needs of ethnic minorities, the programs have not been formally evaluated.

4 The full text of Decision 132 is provided in Appendix A.

5 In the course of our resurvey, we were not able to track down all of the households that were originally surveyed in 2002, and thus not able to construct a “perfect” panel. The attrition was 122 households, or 10 percent of the original sample. We compared the 2002 attributes of our panel households (1,126) with those of the households that we lose to attrition (122). Not conditioning on commune, we lose slightly more non-minorities, households with less annual land, and smaller households with slightly higher incomes. Conditioning on commune, there are no significant differences between the panel households, and the attrited ones (within the sample communes). This suggests that our panel households provide an unbiased picture of the changes between 2002 and 2008 conditional on the commune being re-sampled.

6 It is important to keep in mind the relatively small number of Kinh households in Kon Tum in our sample (23 in total).

7 The total number of participating communes rises to 39 once we include those that implemented either of programs 132 or 134.

8 The household level data on land received through 132 and 134, and results in Appendix C Table 1 reveal the important role of reclaimed land.

9 In Appendix B, we provide detailed breakdowns of the “non-Kon Tum” results by province (Gia Lai, Dak Lak, and Lam Dong) for Table 4, and all subsequent tables.

10 This discussion highlights the potential difficulty of comparing treatment rates using the different data sources. Note, however, that our panel does not show much of a decline in ethnic minority household size between 2002 and 2008. In Kon Tum, average household size decreases from 5.72 to 5.69, well within sampling error. Outside Kon Tum, the decline is slightly larger, from 5.86 to 5.73. This is much smaller than the decline calculated from the Agricultural Censuses (5.57 to 5.22 outside Kon Tum between 2001 and 2006). We, therefore, do not have any evidence in our data to suggest that the programs led to changes in household structure.

11 While not reported in these tables, we also explored linkages between the amount of land that households said that they received from the programs, and predicted eligibility. Conditional on treatment (“Treat 2”), eligible households in Kon Tum received 0.34 hectares of land, compared to 0.49 for the ineligible. Outside Kon Tum, eligible households received 0.29 hectares, compared to 0.23 for those with more than a hectare of land. As with treatment status itself, there is no evidence that the amount of land received was related to predicted eligibility.

12 We have not been able to identify conclusively the reason for the decline in forestry land, though several alternative possible interpretations exist. First, forestry land may have been recorded differently in the 2002 and 2008 surveys, raising the possibility of measurement error. Second, in Kon Tum, forestry land has been under the ownership of state forests since the 1980s, with households often hired as caretakers. Most of the decline in access to forestland, and forestry sideline income, could be because there was a temporary suspension in this program (which has since been re-implemented). This is consistent with interviews we conducted with commune officials in 2010. Third, there may also have been a genuine reduction in forestland.

13 This is consistent with the general observations by Ravallion and van de Walle (2008) concerning the efficiency and high level of activity of land markets in Vietnam in the post-reform era.

14 Baulch et al. (2012) and Dang (2012) use the VHLSS (2002, 2004, and 2006, respectively) to report rising incomes for minority households, combined with a widening gap with the Kinh. They do not show results separately for Kon Tum.

15 The most obvious refinement is to use the intensity of treatment as our measure, i.e., the amount of land received in the program (including zero). This measure tends to better correlate with household changes in land, but is less correlated with changes in income. We attribute this to measurement error: Measures of land treatment are more correlated with self-reported household allocations of land, while the additional “noise” introduced by household recall of the level of treatment yields a poorer measure of treatment than the binary indicator of program participation.

16 We use estimates from the 2002 specification to extend our exploration of which households received land, and especially whether there is any evidence of selection related to productivity. To do this, we calculate residuals (estimated “Total Factor Productivity” (TFP)) at the household level. We then regress treatment status against “TFP” and find no evidence that estimated TFP is correlated with program participation. For both Kon Tum and provinces outside Kon Tum, the coefficient on TFP is negative, and statistically insignificant. If anything, the households are negatively selected—land does not appear to have been given to the most productive farmers.

17 A comparable example is presented by Bazzi et al. 2016, where households in Indonesia were forced (via migration) to switch types of land they farm, and were unable to achieve the same economic returns. There is farm-type specific human capital which may not be portable. The same argument could hold for ethnic minorities in Vietnam, as they are moved from the forest to more sedentary plots.
