# Supplementary material for: Program participation in a targeted land distribution program and household outcomes: evidence from Vietnam
Source: Rev Econ Househ. 2017 Sep 20;16(1):41–74. doi: 10.1007/s11150-017-9390-0 (PMC6407848; doi:10.1007/s11150-017-9390-0)
Supplement: Supplementary file 1 — Supplementary Information [file 11150_2017_9390_MOESM1_ESM.pdf]

Program participation in a targeted land distribution program and household outcomes: Evidence from Vietnam

Review of Economics of the Household

ONLINE APPENDICES

Dwayne Benjamin
University of Toronto
dwayne.benjamin@utoronto.ca

Loren Brandt
University of Toronto
brandt@chass.utoronto.ca

Brian McCaig
Wilfrid Laurier University
bmccaig@wlu.ca

Nguyen Le Hoa
Institute of Policy and Strategy for Agriculture and Rural Development (IPSARD, Hanoi)
nguyenlehoa@gmail.com

August 28, 2017

1 Outline

These appendices provide supporting tables and figures for the main text. In Appendix A, we provide the documents describing Programs 132 and 134; In Appendix B, we show selected results for the Central Highlands provinces outside Kon Tum ("Non-Kon Tum"), broken down by separate provinces (Gia Lai, Dak Lak, and Lam Dong); In Appendix C we report sources of land used for redistribution; and in Appendix D, we explore the decline in sideline income in 2008. More specifically:

- Appendix A: Decision 132;
- Appendix A: Decision 134;
- Appendix B Table 1: Sample Sizes and Treatment Rates by Province;
- Appendix B Table 2: Comparisons of households in 2002 (Minority households outside Kon Tum);
- Appendix B Table 4: "Treatment" Rates by 2002 Land Holdings ("Potential Eligibility"), Provinces Outside Kon Tum;
- Appendix B Table 5: 2002 Land Status and 2008 Reported Treatment Rates by 2002 Per Capita Income Quartile, Provinces Outside Kon Tum;
- Appendix B Table 6: Exploring Linkages Between Potential Eligibility, Other Household Characteristics, and Treatment Status: Can We Predict Treatment Status? (Based on Treat 2) Provinces Outside Kon Tum;
- Appendix B Table 7: Land Outcomes: 2002 versus 2008, Provinces Outside Kon Tum;
- Appendix B Table 8: Changes to Income and Work, 2002-2008 (Panel Households), Provinces Outside Kon Tum;
- Appendix B Table 9: How much crop income can be derived from a hectare of land? OLS Regressions: Various Specifications, Provinces Outside Kon Tum;
- Appendix B Table 10: Estimated Effect of Program Participation on Various Outcomes, Provinces Outside Kon Tum;
- Appendix C Table 1: Land Redistributed by Communes Through 132: By Type and Source;

- Appendix D Table 1: Participation Rates by Component of Agricultural Sideline Income for Ethnic Minority Households, 2002 and 2008;
- Appendix D Table 2: Participation Rates by Component of Forestry Income for Ethnic Minority Households, 2002 and 2008;
- Appendix D Table 3: Mean Per Capita Income from Agricultural Sidelines for Ethnic Minorities in Rural Areas

Appendix A Official Documents Pertaining to Decisions 132 and 134

A.1 Decision 132

Decision 132/2002/QG-TTg dated 08 October 2002 of the Prime Minister on redistributing production and residential land for ethnic minority people in Central Highlands

Article 1. Resolving land for ethnic minority people in Central Highlands, to ensure that the ethnic minority households can have basic land for production activities as well as residential land. This is to improve their lives, enhance the development and ensure the security in Central Highland regions. Land should be basically redistributed to ethnic minority households who have not or lack of residential and production land at the end of 2003.

Article 2. The minimum distribution of agriculture land and residential land for each household is 1 hectare of terrace land or 0.5 hectare of paddy land (single crop) or 0.3 hectare of paddy land (double crop) and 400 m2 for residential land. As for perennial land, basing on production capability and the lack of land situation, there will be a suitable amount of land to be redistributed. The households who have not agriculture land will be provided forestry land, the distributed land amount will be followed the direction of the decree no 163/1999/NDD-CP dated 16 November 1999 of the Government on land redistribution, land lending to organization, households and individuals to use permanently for forestry purpose.

Article 3. Resolving principles

1. Ensure the equality and transparency of the redistribution to households, villages based on

Government regulation and land policy. No consideration on resolving the issue of requesting old land.

2. To be suitable with custom of each ethnic group in close connection with the local socio-economic development plan towards the target of building a civilized and modern rural area, conserving traditional culture of each ethnic group.

3. Land redistributed households should directly managed and utilized their residential and production land. Within 10 years, they are not allowed to sell, mortgage in any form. If they are discovered to do that, they will be withdrawn and be not able to be redistributed again.

Article 4. Land fund for redistributing to ethnic minority households includes:

1. Land from state owned agriculture and forestry farm consists of: a. Excessive land after re-planning, land has not been used or ineffective production land; b. Land is near river wharf or villages where ethnic minority people are living in; c. Land from the households who have contract with state owned agriculture and forestry farm. If the average land holding of these households exceed local general average land holding, it will be adjusted to give the excessive land to the land fund. The land includes annual land, perennial land and forestry land.

2. Withdrawn land from state owned agriculture and forestry farm if necessary

3. Withdrawn land from ineffective production land, wrong purpose use of enterprises or from the enterprises which are closed down.

4. Land from farmers who have large land and would like to transfer a part of their land with compensation following the Government regulation.

5. Land for public use which has been managed by local authority

6. Reclaimed land, unexploited land

7. Forestry land with water source, poor or low economic effectiveness forestry land are allowed to change the using purpose into production land (the implementation of converting

from forestry land to production land following government regulations on land and forestry development and protection is a must.)

Article 5. Implementation budget

1. *Central budget*: Budget for reclaiming is on average of 4 million VND per hectare. As for households who reclaim themselves following local plan, they are allocated the same amount of money. The compensation for withdrawn land from households reclaimed land is no more than 4 million VND/ha.

2. *For withdrawn garden value*

   (a) *State owned enterprises garden*: If the enterprises garden is invested by state budget, then the budget allocated to the enterprise will be noted as reduction correlatively to the practical value of the garden after reassessment and be debited against to household who is allocated the garden. If the enterprise borrows from bank to invest in the garden, they are leaved the debt in a certain time with no interest rate, the state will support the bank in term of interest rate.

   (b) As for withdrawn garden which are from and invested by private enterprises and individuals, then local authority will resolve by their own budget.

3. Households are allocated perennial garden will be debited (with no interest rate) the garden value at the allocated time. Time for repaying debt will be appropriate to the economic cycle and production time left of the garden, but the maximum time is 10 years. Households repay debt before the regulated time will be reduced price. Specific reduction level will be regulated by the Ministry of Finance. The President of People Committees in Central Highlands will preside the withdrawing of garden debts of households who are allocated land relating to garden. Obtained debt, will be firstly paid for the owners of the gardens, and then the rest will be invested to local infrastructure, irrigation, electricity, clean water.

4. Ministry of Finance, Ministry of Planning and Investment will base on specific proposal of each province to approve and arrange enough budgets during two years (2002-2003) to implement. In 2002, state budget will be advanced for the implementation; the rest will be taken from proposed budget in 2003.

Article 6. Form of land redistribution and land use management

1. Production land: Ethnic minority households who depend on agriculture and forestry production have no land or are lack of production land will be distributed land directly from the local people committee with the standard amount following Article 2 in this Decision. In order to help ethnic minority households to have enough land for production ensuring their lives, within 10 years, land distributed households of this program will be not allowed to transfer and mortgage in any form. Any organization or individual come to purchase ethnic minority households land will be seized without compensation. After redistributing land to ethnic minority households, local people committees have the responsibility to monitor carefully the use and management of redistributed land following this Decision in order to avoid the situation of selling or mortgaging land.

2. Residential land: The households who have no residential land will be provided land to construct their houses, the standard amount is indicated in Article 2 of this Decision and will be given land use certificate permanently following government regulations.

Article 7. Implementation organization

1. The President of the Provincial People Committees in Central Highlands will have the responsibility to review land demanding household list of their own provinces and coordinate with Ministry of Agriculture and Rural Development, Ministry of Military, State owned rubber corporation, State owned coffee corporation, Army Corps 15, Army Corps 16 to rearrange state owned agriculture and forestry farms, state owned agriculture enterprises in local area as in following direction: transfer all unexploited land, ineffective land and a part of currently used land (including land is near river wharf and village) of the state owned agriculture and forestry farms to local authority in order to distribute to ethnic minority households). In 2002, the pilot should be completely implemented in some district, villages in order to get experiences and adjust the implementation plan so that in 2003, production and residential land redistribution is basically finished following this decision. In parallel with land redistribution, the extension activities for agriculture, forestry, money lending, product sale, house construction, etc should be well organized to help ethnic minority people to improve their

lives. Inspection on the management, utilization of land in implemented provinces should be taken place in order to prevent and punish any activities on purchasing land, mortgaging land illegally.

2. Ministry of Agriculture and Rural Development will preside and coordinate with two other Ministries including Ministry of Finance, Ministry of Natural Resources and Environment to assess the land support proposals of Central Highlands provinces following this decision prior to the time President of People Committees in Central these provinces approve their own land support proposals.

3. Ministry of Natural Resources and Environment will preside and coordinate with Ministry of Agriculture and Rural Development to monitor the implementation in Central Highland provinces; Ministry of Finance will take the responsibility to guide and check the budget utilization during the implementation of this decision.

Article 8. This decision is in effect since its signed date.

Article 9. Ministers of the following Ministries: Ministry of Agriculture and Rural Development, Ministry of Finance, Ministry of Planning and Investment, Ministry of Natural Resources and Environment, Ministry of Labour, Invalids and Social Affairs and Minister of Central Ethnic Minority Committee, President of State Bank, President of People Committee in following provinces: Lam Dong, Dak Lak, Gia Lai and Kon Tum have the responsibility to implement this decision./.

Prime Minister Phan Van Khai

A.2 Decision 134

Decision No 134 of the Prime Minister on supporting production land, residential land, houses and daily use water for poor ethnic minority households.

Article 1. Implement policies on supporting production land, residential land, housing and daily use water for poor ethnic minority households in associated with other socio-economic program of Government to support ethnic minority households to afford production activities, improve their lives and escape from poverty.

1. Target group: Local ethnic minority households who are permanent residents, poor ethnic minority households who are living based on agriculture and forestry activities. These target groups should be eligible for the program if they have not or are lack of production land or residential land and meet difficulties in terms of houses, daily use water.

2. Principles:

   (a) Support production land, residential land and houses, daily use water directly to poor ethnic minority households

   (b) Ensure the transparency, equality in supporting to each household in every village following Government regulations and policies.

   (c) To be suitable with custom of each ethnic group, region, and conserve the culture character of all ethnic groups, be suitable with practical conditions and be in associated with local plan on socio-economic development.

   (d) Households who are distributed production land, residential land, houses and daily use water should manage and use the supported items directly. This is to ensure production development, living condition improvement and contribute in poverty reduction. In particular case, if supported households (production and residential land) want to move to another province, they should transfer their land use right to the local authority to redistribute to other poor ethnic minority households. If they do not follow this regulation, Government will withdraw with no compensation to redistribute to ethnic minority households who have not land or are lack of land.

Article 2. About policies

1. Production land: The minimum amount of redistributed land for one household includes 0.5 ha of terrace land or 0.25 ha of single paddy crop or 0.15 ha of double paddy crop. Based on local land fund, labour capacity, and number of members in each household and local budget, the provincial people committees may consider and decide to redistribute land to the ethnic minority households with higher amount.

2. Residential land: The minimum amount of redistributed land is 200m2 for each ethnic minority household in rural area. Based on local land fund and budget, the provincial people

committee will consider to redistribute residential land to ethnic minority households with higher amount. The State will have particular policies to support on production and residential land for poor Kho me ethnic group due to the characteristics of Mekong river delta.

3. Housing: Regarding poor ethnic minority households (including Kho me) have no houses or houses are damaged partly, the support will follow the direction People should construct themselves, the State will support and the community will help.
    (a) Central budget will be delivered to support the amount of 5 million VND/household to construct houses. Based on situation and budget, the local authority will put an additional support and encourage the help of the community
    (b) As for the provinces that possess forest planned and annual wood exploiting plan approved, the provincial people committees are allowed to exploit wood under common regulation to support ethnic minority households to construct their houses. The amount of wood to be distributed will be decided by the provincial people committee. It is not allowed to take this advantage to destroy the forest.
4. Support on daily use water
    (a) The ethnic minority households are scattered living in upland, mountain, etc where are difficult to get daily use water, the State will provide an amount of 0.5 ton cement/household from central budget to construct water tank or support the amount of 300. 000 VND/household to sink well or find a daily use water source.
    (b) As for common daily use water building, the State will support 100% from central budget to villages which have above 50% of total households are ethnic minority households, support 50% to the village which have from 20% to below 50% of total households are ethnic minority households. The local daily use water construction must be ensured of the effectiveness and the sustainability.

Article 3. Land fund for redistributing land to ethnic minority households includes:

1. Public land which have been withdrawn by the State following the general planning. Land is allocated with contract to households from State owned agriculture and forestry farms.

2. Land is withdrawn from State owned agriculture and forestry farms due to ineffective use.

3. Land is reclaimed from bared and hilly land or abandoned land

4. Land is withdrawn from state enterprises that use land ineffectively and for wrong purposes or enterprises are closed down; land is withdrawn from individuals who appropriate illegally.

5. Land is being managed and used by State owned agriculture and forestry farms but is used by ethnic minority households long time ago should be adjusted to be reallocated with contract to ethnic households as production land (including the area of perennial crops or forest) to use following general regulation. The specific amount will be decided by provincial people committee.

6. Land is given or transferred from other households voluntarily

7. As for the case of having no land for agriculture production, forestry land will be used to redistribute. The redistributed amount will be followed the Decree no 163/1999/ND-CP dated 16 November 1999 of the Government in redistributing, lending forestry land over organizations, households and individuals for long term use; and regulations of Land Law.

Article 4. Support in creating production and residential land fund

1. Central budget will support to create production and residential land fund, consisting of reclaiming, compensation for withdrawn land, transferred land from other households who have large land with amount of 5 million VND per hectare. Based on local practical situation, the provinces will decide specific regulations.

2. If the state owned agriculture and forestry farms are assigned to organize production activities for ethnic minority households, they are also supported by central budget for reclaiming with amount of 5 million VN per hectare. Moreover, they are also supported capital for constructing roads, electricity network and small irrigation building.

Article 5. Implementing budget

1. Central budget should ensure the expense amounts following regulations of this decision

2. Local budget should contribute the amount of over 20% of total Central budget as well as encourage other legal budget sources in order to implement this policy.

3. The local take the initiative in providing budget for measuring land, issuing land use certificate for ethnic minority households

Article 6. Implementing organization

1. Provincial people committees have the responsibility to directly and comprehensively preside and organize the implementation of this policy

   (a) Announce publicly the criteria, target group and conduct a survey to obtain the list of poor ethnic households who have not or are lack of production and/or residential land and meet difficulties in terms of housing, or daily use water.

   (b) Design and approve proposals on redistributing production land, residential land, housing, daily use water for poor ethnic minority households in local area (including the promulgation of Decisions on adjusting contracted land and withdrawn land from state owned agriculture and forestry farms which are under the management of ministries and other local situated institutions) in order to send to Ministry of Planning and Investment, Ministry of Finance for reviewing and submitting to the Prime Minister so that annual plans can be approved. The above mentioned activities should be completed during the third quarter of 2004. If there are any constraints, it is needed to report to the Prime Minister and Ministries as well as related institutions to resolve.

   (c) Direct the related institutions, local authorities and other socio-political organizations to implement effectively support policies to poor ethnic minority households, contribute in poverty reduction and improve the lives of ethnic minority households.

   (d) Monitor regularly the implementation process, ensure that the policy go to all ethnic minority households, the corruption is not allowed.

   (e) By the end of 2006, the implementation of policies in this Decision should be basically finished.

2. Ministry of Agriculture and Rural Development has the responsibility in leading, guiding and helping the provinces in constructing and upgrading small irrigation building, supporting on

seeds, animals, resolving daily use water and rural environment hygiene towards the direction of production development and poverty reduction.

3. Ministry of Construction will guild and monitor the provinces in implementing the policies of supporting houses for ethnic minority households.

4. Based on the proposal on resolving production land, residential land, housing, daily use water approved by the provincial people committee, Ministry of Planning and Investment will preside and coordinate with Ministry of Finance to review the plan, allocate additional targeted budget to the provinces within proposed plan and central budget in 2005, 2006 and then submit to the Prime Minister for final decision.

5. Ministry of Finance will submit to the Prime Minister for approval specific policies in terms of withdrawing production land from state owned agriculture and forestry farms (including perennial crops and planted forest) in order to allocate with contract to poor ethnic minority households.

6. Central ethnic minority committee will preside and coordinate with other Ministries, related institutions to guide and monitor the implementation of this Decision, and report to the Prime Minister periodically.

7. Ministries, related institutions based on their own mandates will have the responsibility to monitor and support the provinces to implement effectively the policies regulated in this Decision.

Article 7. This Decision will be in effect after 15 days from the first day to be public in Government legal document. To declare off the Decision no 154/2002/QD-TTg dated 12 November 2002 of the Prime Minister on policies for ethnic minority households and other households in Central Highlands selected for the policies on purchasing houses with deferred payment.

Article 8. Ministers, Heads of the institutions are at the same level with the Ministries, Heads of Governmental institutions and the Presidents of City/Provincial people committees under central management will have the responsibility to implement this Decision./.

Prime Minister Phan Van Khai. Signed.

Appendix B Results by Province Outside Kon Tum

In this appendix we provide further detail in support of the main tables in the text. In particular, we disaggregate the results for minorities "Outside Kon Tum" into those for the separate provinces (Gia Lai, Dak Lak, and Lam Dong). Except for Appendix B Table 1, which shows sample sizes, the remaining tables are numbered to correspond to the ones in the main text.

Appendix C Land Redistributed by Communes Through 132: By Type and Source

This appendix is comprised of a single table that shows the primary sources of land used for redistribution.

Appendix D Agricultural Sideline Income

In this appendix, we investigate the decrease in income from agricultural sidelines among ethnic minority households in Kon Tum in our sample. We show that there have been significant changes in participation among households and compare our results with nationally representative survey data. Our results suggest that the decrease in forestry land for ethnic minorities household in Kon Tum was associated with a significant decline in the share of households participating in various agricultural sideline activities, particularly those related to forestry. This suggests that the decline in sideline income was driven largely by loss of access to forest land in 2008.

D.1 Changes in participation rates within agricultural sidelines

Appendix D Table 1 displays the share of ethnic minority households by province in our sample engaged in the various subcomponents of agricultural sidelines: livestock, agricultural services, forestry, hunting and trapping, and aquaculture. While we focus on Kon Tum, since the fall in agricultural sidelines income is greatest there (see Table 8), we report the participation rates for the other provinces in our sample for comparison purposes.

In 2002, the vast majority of ethnic minority households in Kon Tum in our sample are engaged in livestock, forestry (all of them), hunting and trapping, and aquaculture. Very few of

these households are engaged in agricultural services. However, by 2008, there is a sharp drop in participation rates across livestock, hunting and trapping, and aquaculture within Kon Tum. This suggests that the loss in forestry land within Kon Tum (see Table 7) significantly affected the prevalence of various income-generating activities. Interestingly, within Kon Tum, there is only a minor decrease in the participation rate within forestry. This is notable since forestry accounted for over 50% of agricultural sideline income in Kon Tum in 2002 in our sample. The results for Kon Tum confirm that there was a discrete drop in access to sideline earning activities, as opposed to a decline in returns to these activities. Across the other Central Highlands provinces, there are also some significant changes in participation rates, both increases and decreases, but none of the changes are as large as in Kon Tum. This is consistent with smaller changes in land holdings amongst ethnic minorities in Central Highlands provinces other than Kon Tum (see Table 7).

D.2 Changes in participations rates within forestry

Our questionnaire allows us to disaggregate forestry income into various related activities. In Appendix D Table 2, we show the participation rates for ethnic minority households in our sample by province and year for each sub-activity. We disaggregate forestry income due to its importance in agricultural sideline income for Kon Tum ethnic minorities in 2002 (over 50%). Despite the small drop in forestry participation noted in Appendix D Table 1, there were appreciable changes in participation in various forestry sub-activities.

In 2002, participation in Kon Tum is concentrated in wood; trees for breeding; bamboo; trees for wood; other forestry services; and other trees. Many of these activities essentially disappeared by 2008. These households are no longer involved in trees for wood, bamboo, other trees, and other forestry services. Similarly, there was a large drop in the share of these households involved in tree breeding. The only activity seemingly unaffected was the gathering of wood. Hence, despite little change in the participation rate for aggregate forestry, numerous forestry activities dramatically decreased within our sample of ethnic minority households in Kon Tum. This is consistent with the reduction in forestry land reported in Table 7 and the decline in agricultural sideline income in Table 8. In contrast, participation in the various forestry activities did not decrease as sharply in the other provinces and actually increased for some activities within Lam Dong. Again, this confirms our hypothesis that a loss of access to forest land (even if transitory) drove the decline

in sideline income.

D.3 Changes in income from agricultural sidelines

While we do not know the specific reason for the reduction in forestry land and associated income for ethnic minorities within Kon Tum, we can say a little more about the timing of this occurring between 2002 and 2008 by turning to an alternative data source. Benjamin, Brandt, and McCaig (2017) provide income estimates using the 2002, 2004, 2006, and 2008 VHLSS's. These surveys are representative by rural and urban areas within provinces. Note that these estimates will include households from communes that are not part of our sample.

In Appendix D Table 3, we report estimates of real mean per capita agricultural sideline income by province-year for ethnic minorities in rural areas of our four provinces. Focusing on Kon Tum, we see an increase in real income from agricultural sidelines between 2002 and 2004, followed by decreases in 2006 and 2008. This suggests that the reduction in forestry land and the loss of associated income occurred largely between 2006 and 2008. While the decline mirrors the pattern in our data, the 15% drop is not quite as great as reported in our sample in Table 8. Outside of Kon Tum, sideline income fluctuates in Gia Lai, increase in DaK Lak, and increases then falls precipitously in Lam Dong.

Appendix B Table 1: Sample Sizes and Program Participation (Treatment Rates) By Province

Province	Communes	Treated Communes	Households	Treated Households	Treatment Rate (%)
Kon Tum	9	7	207	36	17.4
Gia Lai	14	3	277	3	1.1
Dak Lak	13	3	229	11	4.8
Lam Dong	7	2	123	9	7.3
Overall	43	15	836	59	7.1

Notes: 1) Source: CHVLSS 2008; 2) Treatment rates are based on the "Treat 2" definition, where households report participation in either Program 132 or Program 134.

Appendix B Table 2: Comparisons of Ethnic Minority Households Households Outside Kon Tum in 2002

	Gia Lai	Dak Lak	Lam Dong
**Average Household Land**			
Annual Land (Ha.)	1.06	0.56	0.46
Perennial Land (Ha.)	0.23	0.69	0.57
Agricultural Land (Annual+Perennial, Ha.)	1.30	1.25	1.04
Forestry Land (Ha.)	0.05	0.01	0.04
**Land Distribution**			
Proportion of Households with:			
Annual Land = 0	0.01	0.23	0.31
Annual Land > 0 & < 0.5	0.21	0.32	0.33
Annual Land ≥0.5 & < 1.0	0.30	0.23	0.19
Annual Land ≥ 1.0	0.48	0.22	0.17
Agricultural Land = 0	0.01	0.01	0.01
Agricultural Land > 0 & < 0.5	0.14	0.15	0.25
Annual Land ≥ 0.5 & < 1.0	0.25	0.27	0.27
Agricultural Land ≥ 1.0	0.60	0.57	0.47
**Household Income and Composition**			
Household Income	14,459	16,684	16,169
Crop Income	6,764	11,076	8,557
Sidelines	3,057	1,799	2,609
Wages	3,487	2,514	3,718
Family Business	337	208	534
Other Income	464	635	183
Remittances	351	453	568
Per Capita Income	2,600	3,046	2,831
**Simple Demographics**			
Household Size	5.83	5.86	5.92
Maximum Male Education	4.16	5.00	4.16
Maximum Female Education	2.73	4.28	3.08
**Household Labour (Days per year)**			
Male, Days in Farming	200	196	270
Male, Days in Non-Farm work	9	7	11
Female, Days in Farming	228	193	234
Female, Days in Non-Farm work	3	4	35
**Main Ethnic Groups (%)**			
Xo Dang (Sedang)		8	
Ba Na (Bahnar)	25		
Nung		11	
Churu			16
Ngai	69		
ÃŁ De (Rhade)		61	
Thai	3		
La Ha			1
Co Ho			83
N	277	229	267

Notes: 1) Source: VHLSS 2002; 2) Household education is the years of education of the highest educated (male or female) adult in the household. This is calculated for household members 15 and older. If there is no male or female older than 15, the maximum is calculated as "zero"; 3) Income variables are expressed in '000 VND (2007 prices); 4) Land Distribution based on households with land by size (in hectares) in the household.

Appendix B Table 4: Treatment Rates by 2002 Land Holdings: Provinces Outside Kon Tum

**Various Definitions of Treatment Status**								
**Household-Level Reports of Program Participation**								
		**Potentially Eligible (Based on initial land holdings, (Ha.))**				**"Inelig-ible"**	**F-Tests (p-values)**	
**Gia Lai**	All	0	0 to 0.5	0.5 to 1.0	< 1.0	≥ 1.0	OLS	FE
Treat 1: 132 Indicator (%)	0.0	0.0	0.0	0.0	0.0	0.0		
Treat 2: 132 or 134 Indicator (%)	1.1	0.0	5.1	1.4	2.7	0.0	1.58 (0.24)	1.19 (0.35)
Treat 3: 132, 134, or Reclaimed (%)	12.6	50.0	12.8	7.1	9.9	14.5	0.94 (0.45)	0.79 (0.52)
Sample Size	277	2	39	70	111	166	277	277
Proportion of Households (%)	100	0.7	14.1	25.3	40.1	59.9		
**Dak Lak**	All	0	0 to 0.5	0.5 to 1.0	< 1.0	≥ 1.0	OLS	FE
Treat 1: 132 Indicator (%)	3.9	0.0	0.0	0.0	0.0	6.9	1.44 (0.25)	0.62 (0.62)
Treat 2: 132 or 134 Indicator (%)	4.8	0.0	0.0	3.3	2.0	6.9	1.19 (0.34)	1.11 (0.38)
Treat 3: 132, 134, or Reclaimed (%)	6.6	0.0	0.0	6.6	4.0	8.	1.50 (0.26)	0.98 (0.44)
Sample Size	229	3	35	61	99	130	229	229
Proportion of Households (%)	100	1.3	15.3	26.6	43.2	56.8		
**Lam Dong**	All	0	0 to 0.5	0.5 to 1.0	< 1.0	≥ 1.0	OLS	FE
Treat 1: 132 Indicator (%)	3.3	0.0	0.0	6.1	3.1	3.4	0.65 (0.56)	0.44 (0.66)
Treat 2: 132 or 134 Indicator (%)	7.3	0.0	6.5	12.1	9.2	5.2	1.73 (0.26)	1.12 (0.38)
Treat 3: 132, 134, or Reclaimed (%)	9.8	0.0	9.7	15.2	12.3	6.9	1.79 (0.25)	0.10 (0.90)
Sample Size	123	1	31	33	65	58	123	123
Proportion of Households (%)	100	0.8	25.2	26.8	52.8	47.2		

Notes:1) Source: VHLSS 2002 and CHVLSS 2008; 2) Treatment status is based on household reports from the CHVLSS 2008; 3) "Treat 1", "Treat 2", and "Treat 3" refer to different potential definitions of self-reported treatment status, depending on household responses to questions pertaining to participation. 4) Potential Eligibility is based on reported land holdings in 2002 (combined annual plus perennial land). The column "All" refers to all minority households in the province, and gives the overall "treatment rate."; 5) All calculations based on sample of minority households only; 6) F-Tests test whether there is a correlation between treatment status and being in a "potential eligibility" category. This is based on a regression of treatment status on indicator variables for initial landholdings being in the zero, zero to 0.5, or 0.5 to 1.0 categories. The F-tests are computed with ("FE"), and without ("OLS") commune dummies; 8) Robust F-statistics calculated with Variance-Covariance clustered at the commune level; 7) Statistically significant F-statistics (at the 5% level), highlighted in bold italics.

Appendix B Table 5: 2002 Land Status and 2008 Reported Treatment Rates by 2002 Per Capita Income Quartile: Provinces Outside Kon Tum

	Means by Income Quartile				F-Tests (p-values)	
	Q1	Q2	Q3	Q4	OLS	FE
**Gia Lai**						
Per Capita Household Income (2002)	1,359	2,278	2,895	5,093	***215 (0.00)***	***207 (0.00)***
Annual Land in 2002 (Ha.)	0.94	1.03	1.05	1.36	0.86 (0.48)	2.80 (0.08)
Perennial Land in 2002 (Ha.)	0.11	0.16	0.34	0.51	***3.77 (0.04)***	***5.36 (0.01)***
Total Agricultural Land in 2002 (Ha.)	1.05	1.18	1.39	1.87	***4.19 (0.03)***	***9.55 (0.00)***
Potentially Eligible (Total land < 1.0 Ha.)	0.45	0.41	0.37	0.31	2.04 (0.16)	***6.60 (0.01)***
Treat 1: 132 Indicator (%)	0.0	0.0	0.0	0.0		
Treat 2: 132 or 134 Indicator (%)	0.0	2.1	2.3	0.0	1.77 (0.21)	1.25 (0.33)
Treat 3: 132, 134, or Reclaimed (%)	12.5	12.6	14.0	11.8	0.03 (0.99)	0.48 (0.70)
**Dak Lak**						
Per Capita Household Income (2002)	1,435	2,268	2,993	5,327	***234 (0.00)***	***70 (0.00)***
Annual Land in 2002 (Ha.)	0.58	0.44	0.47	0.75	***4.34 (0.03)***	1.67 (0.23)
Perennial Land in 2002 (Ha.)	0.29	0.61	0.92	0.88	***4.32 (0.03)***	***3.77 (0.04)***
Total Agricultural Land in 2002 (Ha.)	0.87	1.05	1.39	1.62	***4.81 (0.02)***	***4.80 (0.02)***
Potentially Eligible (Total land < 1.0 Ha.)	0.59	0.50	0.37	0.29	***4.83 (0.02)***	2.84 (0.08)
Treat 1: 132 Indicator (%)	4.9	0.0	7.5	1.7	0.76 (0.54)	0.75 (0.54)
Treat 2: 132 or 134 Indicator (%)	4.9	2.4	9.0	1.7	0.38 (0.77)	0.59 (0.64)
Treat 3: 132, 134, or Reclaimed (%)	4.9	7.1	10.4	3.4	0.57 (0.64)	0.04 (0.99)
**Lam Dong**						
Per Capita Household Income (2002)	1,445	2,292	2,884	5,013	***83.74 (0.00)***	***63 (0.00)***
Annual Land in 2002 (Ha.)	0.26	0.40	0.53	0.70	1.15 (0.40)	3.30 (0.10)
Perennial Land in 2002 (Ha.)	0.55	0.42	0.60	0.73	0.85 (0.51)	***7.59 (0.02)***
Total Agricultural Land in 2002 (Ha.)	0.81	0.82	1.13	1.43	1.80 (0.25)	***8.26 (0.02)***
Potentially Eligible (Total land < 1.0 Ha.)	0.70	0.61	0.49	0.30	2.48 (0.16)	***10.48 (0.01)***
Treat 1: 132 Indicator (%)	3.0	0.0	5.7	3.7	0.42 (0.74)	0.43 (0.74)
Treat 2: 132 or 134 Indicator (%)	12.1	7.1	5.7	3.7	1.45 (0.32)	0.52 (0.69)
Treat 3: 132, 134, or Reclaimed (%)	15.2	10.7	8.6	3.7	3.29 (0.10)	1.78 (0.25)

Notes: 1) Source: VHLSS 2002 and CHVLSS 2008; 2) This table reports means for 2002, and 2008 reported treatment status, outcomes by per capita income quartile groups (calculated on the basis of 2002 per capita income); 3) The per capita income quartiles are calculated over the entire Central Highlands (ethnic minority households only); 4) All statistics calculated over ethnic minority households only; 5) F-Tests test whether there is a correlation between a given variable (e.g., income, land, or treatment status) and being in an income quartile. 6) The F-tests are computed with ("FE"), and without ("OLS") commune dummies; 8) Robust F-statistics calculated with Variance-Covariance clustered at the commune level; 7) Statistically significant F-statistics (at the 5% level), highlighted in bold italics.

Appendix B Table 6: Exploring Linkages Between Potential Eligibility, Other Household Characteristics, and Treatment Status: Can We Predict Treatment Status? (Based on Treat 2)

	Provinces Outside Kon Tum					
	**Gia Lai**		**Dak Lak**		**Lam Dong**	
	**OLS**	**FE**	**OLS**	**FE**	**OLS**	**FE**
F-Annual Land Categories	0.973	1.281	0.618	1.907	0.577	3.083
	(0.435)	(0.322)	(0.617)	(0.182)	(0.651)	(0.112)
F Perennial Land Categories	1.293	0.293	2.047	2.183	0.846	1.269
	(0.318)	(0.830)	(0.161)	(0.143)	(0.517)	(0.367)
F Forestry Land Categories	1.422	1.593	0.691	***5.224***	***211.805***	***976.720***
	(0.276)	(0.241)	(0.520)	***(0.023)***	***(0.000)***	***(0.000)***
F ALL Land Categories	0.684	0.519	2.708	***63.802***	***472.550***	***1167.430***
	(0.699)	(0.822)	(0.058)	***(0.000)***	***(0.000)***	***(0.000)***
F Household Characteristics	0.781	0.949	1.875	0.944	2.625	2.338
	(0.557)	(0.467)	(0.180)	(0.472)	(0.140)	(0.169)

Notes: 1) This table reports F-statistics ofor the joint significance of household variables of a given type, in a regression explaining household reported treatment status; 2) Household characteristics include: log per capita household income in 2002, household size in 2002, and maximum male and female education in 2002; 3) The F-tests are computed with ("FE"), and without ("OLS") commune dummies; 4) Robust F-statistics calculated with Variance-Covariance clustered at the commune level; 5) Statistically significant F-statistics (at the 5% level), highlighted in bold italics.

Appendix B Table 7: Land Outcomes: 2002 versus 2008 (Ethnic Minority Households Outside Kon Tum)

	**Gia Lai**		**Dak Lak**		**Lam Dong**	
	2002	2008	2002	2008	2002	2008
Average Annual Land (Ha.)	1.06	1.61	0.56	0.54	0.46	0.37
Average Perennial Land (Ha.)	0.23	0.31	0.69	0.76	0.57	0.64
Average Agricultural Land (Ha.)	1.30	1.92	1.25	1.31	1.04	1.01
Average Forestry Land (Ha.)	0.05	0.08	0.01	0.00	0.04	0.00
**Percentage of households:**						
Total Equal to 0	0.7	0.4	1.3	5.2	0.8	4.1
Total Less than 0.5 Ha.	14.1	11.2	15.3	9.2	25.2	23.6
Total Less than 1.0 Ha.	40.1	25.3	43.2	44.5	52.8	51.2

Notes: 1) Source: VHLSS 2002 and CHVLSS 2008; 2) Land variables are expressed as average hectares per household; 3) The percentages of household with land below a particular cutoff is also the "Cumulative Distribution Function," (or CDF) of the land distribution.

Appendix B Table 8: Changes to Income and Work, 2002-2008 (Panel Households): Ethnic Minority Households Outside Kon Tum

	**Gia Lai**		**Dak Lak**		**Lam Dong**	
	2002	2008	2002	2008	2002	2008
**Income:**						
Per Capita Income	2,600	4,595	3,046	6,733	2,831	5,545
Per Capita Expenditure	1,819	3,240	2,489	3,814	2,345	3,814
Log per capita income	7.73	8.25	7.88	8.45	7.84	8.43
Log per capita expenditure	7.44	7.96	7.74	8.11	7.65	8.15
Household Income	14,459	24,031	16,684	36,209	16,169	29,719
Crop Income	6,764	13,268	11,076	28,513	8,557	18,115
Sidelines	3,057	3,313	1,799	1,240	2,609	2,392
Wages	3,487	5,903	2,514	3,905	3,718	5,017
Family Business	337	157	208	455	534	621
Other Income	464	644	635	283	183	3,096
Remittances	351	746	453	1,814	568	479
**Labour (Days worked):**						
Male, Days in Farming	200	228	196	211	270	216
Male, Days in Non-Farm work	9	16	7	14	11	15
Female, Days in Farming	228	226	193	195	234	183
Female, Days in Non-Farm work	3	7	4	11	35	26

Notes: 1) Source: VHLSS 2002 and CHVLSS 2008 (Panel Households); 2) All values are expressed in constant '000 VND (2008 Survey prices)

Appendix B Table 9: How much crop income can be derived from a hectare of land?OLS Regressions: Various Specifications for Provinces Outside Kon Tum

	(1)	(2)	(3)	(4)
Output Measure:	2002 Level	2008 Level	Change	Change
Land Measure:	2002 Level	2008 Level	2002 Level	Change
**Gia Lai:**				
Annual Land (Ha.)	1,340	***3,785***	937	1,750
	(890)	***(879)***	(809)	(1361)
Perennial Land (Ha.)	***3,288***	***5,162***	-396	***3,705***
	(963)	***(1355)***	(1191)	***(852)***
**Dak Lak:**				
Annual Land (Ha.)	***4,574***	8,566	5,422	4,972
	(644)	(4976)	(4576)	(3105)
Perennial Land (Ha.)	***7,952***	***20,437***	1,564	13,035
	(1582)	***(5744)***	(2579)	(10115)
**Lam Dong:**				
Annual Land (Ha.)	***6,502***	***8,539***	1,620	***11,101***
	(1395)	***(2475)***	(2221)	***(2703)***
Perennial Land (Ha.)	***6,427***	***18,663***	3,742	***10,948***
	(998)	***(3929)***	(5641)	***(2655)***

Notes: 1) Source: VHLSS 2002 and CHVLSS 2008 (Panel); 2) Each column represents a regression of crop income on land holdings (annual and perennial); 3) For crop income: specifications include the 2002 level, 2008 level, and the change between 2008 and 2002; 4) Land holdings are measured as either: the 2002 level, the 2008 level, or the change between 2002 and 2008; 5) All specifications include controls for commune fixed effects, household size, and education; 6) Robust standard errors in parentheses, cluster-corrected at the commune-level, and statistically significant coefficients (5%) in bold italics; 7) The regressions are estimated separately for each subsample.

Appendix B Table 10: Estimated Effect of Program Participation on Various Outcomes (Provinces Outside Kon Tum)

	Annual Land (Ha.)	Perennial Land (Ha.)	Total Land (Ha.)	Household Income (VND)	Crop Income (VND)	Sideline Income (VND)	Male Days Farming (Days)	Female Days Farming (Days)	Total Days Farming (Days)
**Effect for Kon Tum**	0.28	-0.04	0.24	***2954***	***2690***	-72	21.1	49.6	70.7
	(0.18)	(0.14)	(0.18)	***(894)***	***(941)***	(177)	(20.3)	(31.4)	(39.9)
**Effect for CH Outside Kon Tum**	-0.3	0.07	-0.23	-4615	-1873	***-970***	16.3	-27.3	-11.0
	(0.16)	(0.18)	(0.21)	(2424)	(2148)	***(432)***	(43.2)	(37.0)	(76.7)
**Gia Lai:**	-0.43	***-0.31***	-0.74	-1,748	-112	-586	22	2	24.2
	(0.49)	***(0.15)***	(0.42)	(5,525)	(3,881)	(1,138)	(59)	(133)	(174.6)
**Dak Lak**	***-0.30***	-0.01	-0.31	-4,254	-3,996	-109	***-63***	-49	***-111.5***
	(0.14)	(0.32)	(0.23)	(6,390)	(5,370)	(229)	***(23)***	(26)	***(33.8)***
**Lam Dong**	***0.17***	0.49	***0.66***	4,760	6,584	-1,198	***108***	20	***128.5***
	(0.08)	(0.29)	***(0.24)***	(6,496)	(5,412)	(613)	***(32)***	(28)	***(48.0)***

Notes: 1) Source: VHLSS 2002 and CHVLSS 2008 (Panel), Minority Households Only; 2) Each reported coefficient is the regression coefficient on a measure of treatment status from a regression of a change in household outcomes on treatment status (Based on "Treat 2"), and covariates; 3) All specifications include commune fixed effects, flexible controls for household land endowments in 2002, household size and education in 2002, log pcy in 2002, and a complete set of initial measures of the 2002 dependent variables in this table; 4) Robust standard Errors in parentheses (Cluster-corrected at the commune-level), and statistically significant coefficients (5%) in bold italic.

Appendix C Table 1: Land Redistributed by Communes Through 132: By Type and Source

		Land Source:					
		**1**	**2**	**3**	**4**	**Total**	
	**Type of Land:**	**Amount (Ha.)**	**Amount (Ha.)**	**Amount (Ha.)**	**Amount (Ha.)**	**Amount (Ha.)**	**% of Total**
**Kon Tum:**	Annual	644.0	141.5	191.7	0.0	977.3	90.2
	Perennial	0.0	0.0	0.0	0.0	0.0	0.0
	Forest	0.0	0.0	0.0	0.0	0.0	0.0
	Unused	0.0	0.0	106.1	0.0	106.1	9.8
	Other	0.0	0.0	0.0	0.0	0.0	0.0
	**ALL**	644.0	141.5	297.8	0.0	1083.4	100.0
	% of Total	59.4	13.1	27.5	0.0	100.0	
**Non-Kon Tum**	Annual	64.3	196.5	74.6	13.1	348.6	53.1
	Perennial	193.7	5.5	0.0	45.3	244.4	37.2
	Forest	0.0	0.0	0.0	0.0	0.0	0.0
	Unused	0.0	6.6	38.7	16.5	61.8	9.4
	Other	1.9	0.0	0.0	0.0	1.9	0.3
	**ALL**	259.9	208.6	113.3	74.9	656.8	100.0
	% of Total	39.6	31.8	17.3	11.4	100.0	

Notes: 1) Source: CHVLSS 2008 (Commune Survey); 2) Land Sources:

1. Land transferred from state owned farms and forestry plantations
2. Land from other households with compensation
3. Reclaimed land
4. Other

Appendix D Table 1: Participation Rates by Component of Agricultural Sideline Income for Ethnic Minority Households, 2002 and 2008

	Kon Tum		Gia Lai		Dak Lak		Lam Dong	
	**2002**	**2008**	**2002**	**2008**	**2002**	**2008**	**2002**	**2008**
Livestock	0.976	0.536	0.646	0.589	0.725	0.655	0.455	0.512
Agricultural Services	0.019	0.000	0.040	0.015	0.057	0.009	0.049	0.032
Forestry	1.000	0.957	0.949	0.949	0.467	0.198	0.756	0.832
Hunting & Trapping	0.793	0.115	0.022	0.102	0.048	0.043	0.000	0.008
Aquaculture	0.923	0.316	0.433	0.258	0.258	0.168	0.073	0.008

Notes: 1) Source: VHLSS 2002 and CHVLSS 2008; 2) The table reports the share of ethnic minority households in our panel dataset engaged in each agricultural sideline activity.

Appendix D Table 2: Participation Rates by Component of Forestry Income for Ethnic Minority Households, 2002 and 2008

	Kon Tum		Gia Lai		Dak Lak		Lam Dong	
	**2002**	**2008**	**2002**	**2008**	**2002**	**2008**	**2002**	**2008**
Mu oil trees	0.000	0.005	0.000	0.007	0.000	0.000	0.000	0.000
Cinnamon	0.000	0.000	0.000	0.000	0.000	0.000	0.000	0.000
Anise	0.000	0.000	0.000	0.000	0.000	0.000	0.000	0.000
Pine	0.000	0.000	0.000	0.000	0.000	0.000	0.000	0.024
Varnish trees	0.000	0.000	0.000	0.000	0.000	0.000	0.008	0.000
Trees for wood	0.340	0.005	0.047	0.069	0.026	0.004	0.056	0.000
Bamboo	0.426	0.005	0.164	0.000	0.073	0.004	0.072	0.008
Fan palm trees	0.000	0.000	0.000	0.000	0.000	0.000	0.000	0.000
Water coconut	0.000	0.000	0.000	0.000	0.000	0.000	0.000	0.000
Other trees	0.153	0.000	0.120	0.080	0.034	0.004	0.096	0.256
Wood	0.995	0.933	0.949	0.935	0.470	0.198	0.640	0.656
Forest plantation, protection, maintenance, improvement	0.134	0.014	0.004	0.004	0.000	0.000	0.096	0.008
Trees for breeding and other products collected from the forest	0.990	0.589	0.716	0.273	0.159	0.000	0.376	0.496
Other forestry services	0.321	0.000	0.004	0.004	0.000	0.000	0.000	0.232

Notes: 1) Source: VHLSS 2002 and CHVLSS 2008; 2) The table reports the share of ethnic minority households in our panel dataset engaged in each forestry related activity.

Appendix D Table 3: Mean Per Capita Income from Agricultural Sidelines for Ethnic Minorities in Rural Areas

	**2002**	**2004**	**2006**	**2008**
Kon Tum	974	1,274	964	838
Gia Lai	791	657	479	622
Dak Lak	504	465	601	763
Lam Dong	474	728	725	164

Notes: 1) The sample is all ethnic minority households in the indicated provinces in the 2002, 2004, 2006, and 2008 VHLSS's. 2) The income estimates are derived from the dataset in Benjamin, Brandt, and McCaig (2017); 3) All estimates are weighted by sampling weights; 4) All values are in thousands of Vietnamese dong in 2012 prices.
